# Supplementary material for: CuI-Catalyzed Coupling Reactions of 4-Iodopyrazoles and Alcohols: Application toward Withasomnine and Homologs
Source: Molecules. 2021 Jun 2;26(11):3370. doi: 10.3390/molecules26113370 (PMC8199780; doi:10.3390/molecules26113370)

Suplementaly Materials

CuI-Catalyzed Coupling Reactions of 4-Iodopyrazoles and Alcohols: Application toward Withasomnine and Homologs

Yoshihide Usami *, Yumika Kubo, Toshiki Takagaki, Nao Kuroiwa, Jun Ono, Kohei Nishikawa, Ayaka Nakamizu, Yuya Tatsui, Shinya Harusawa, Noboru Hayama and Hiroki Yoneyama

Department of Pharmaceutical Organic Chemistry, Osaka University of Pharmaceutical Sciences, 4-20-1 Nasahara, Takatsuki, Osaka 569-1094, Japan; e12343@gap.oups.ac.jp (Y.K.); e16442@gap.oups.ac.jp (T.T.); e16332@gap.oups.ac.jp (N.K.); e15439@gap.oups.ac.jp (J.O.); e15637@ga@.oups.ac.jp (K.N.); e14519@gap.oups.ac.jp (A.N.); e18902@gap.oups.ac.jp (Y.T.); harusawa@gly.oups.ac.jp (S.H.); hayama@gly.oups.ac.jp (N.H.); yoneyama@gly.oups.ac.jp (H.Y.).

* Correspondence: usami@gly.oups.ac.jp; Tel.: +81-726-90-1087

Contents

Figure S1,2. ^{1}H- and ^{13}C-NMR spectra of **4c** -3
Figure S3,4. ^{1}H- and ^{13}C-NMR spectra of **4d** -4
Figure S5,6. ^{1}H- and ^{13}C-NMR spectra of **4e** -5
Figure S7,8. ^{1}H- and ^{13}C-NMR spectra of **4f** - 6
Figure S9,10. ^{1}H- and ^{13}C-NMR spectra of **4g** -7
Figure S11,12. ^{1}H- and ^{13}C-NMR spectra of **4i** -8
Figure S13,14. ^{1}H- and ^{13}C-NMR spectra of **4k** -9
Figure S15,16. ^{1}H- and ^{13}C-NMR spectra of **4l** -10
Figure S17,18. ^{1}H- and ^{13}C-NMR spectra of **4m** -11
Figure S19,20. ^{1}H- and ^{13}C-NMR spectra of **4n** -12
Figure S21,22. ^{1}H- and ^{13}C-NMR spectra of **4o** -13
Figure S23,24. ^{1}H- and ^{13}C-NMR spectra of **4r** -14
Figure S25,26. ^{1}H- and ^{13}C-NMR spectra of **4t** -15
Figure S27,28. ^{1}H- and ^{13}C-NMR spectra of **2c** -16
Figure S29,30. ^{1}H- and ^{13}C-NMR spectra of **12** -17
Figure S31. ^{1}H-NMR spectrum of (*Z*)**-13** -18
Figure S32. ^{13}C-NMR spectrum of (*Z*)**-13** -19
Figure S33. ^{1}H-NMR spectrum of (*E*)**-13** - 20
Figure S34. ^{13}C-NMR spectrum of (*E*)**-13** -21
Figure S35,36. ^{1}H- and ^{13}C-NMR spectra of **14** -22
Figure S37,38. ^{1}H- and ^{13}C-NMR spectra of **2d** -23

Figure S39,40. ^{1}H- and ^{13}C-NMR spectra of **16** -24
Figure S41,42. ^{1}H- and ^{13}C-NMR spectra of **17** -25
Figure S43,44. ^{1}H- and ^{13}C-NMR spectra of **18** -26
Figure S45,46. ^{1}H- and ^{13}C-NMR spectra of **19** -27
Figure S47,48. ^{1}H- and ^{13}C-NMR spectra of **15** -28

Figure S1. ^{1}H-NMR spectrum of **4c** (400 MHz, $CDCl_3$)

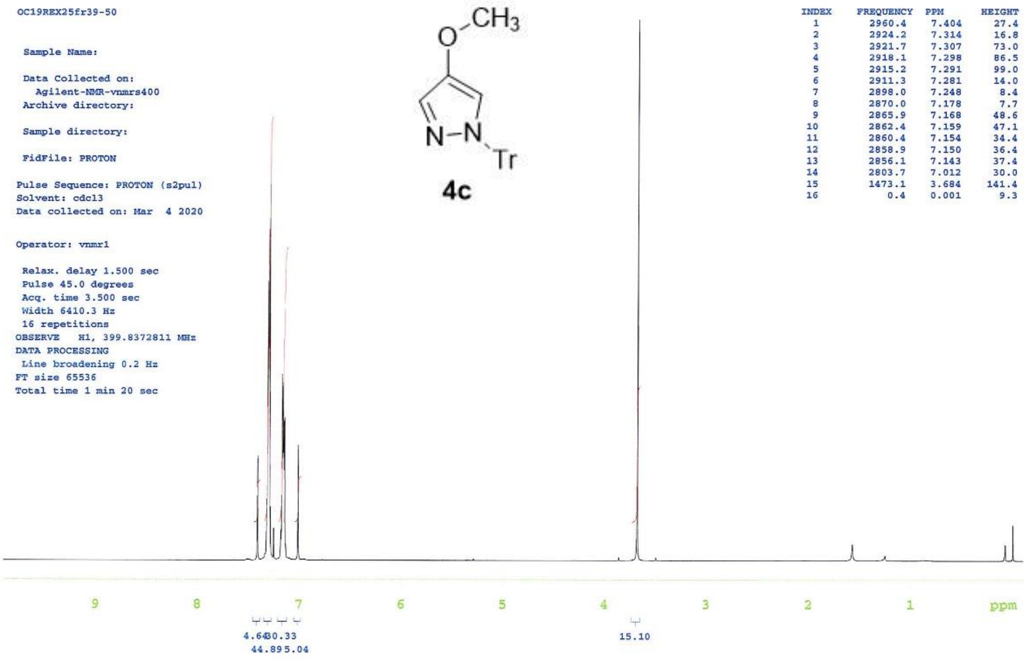

Figure S2. ^{13}C-NMR spectrum of **4c** (100 MHz, $CDCl_3$)

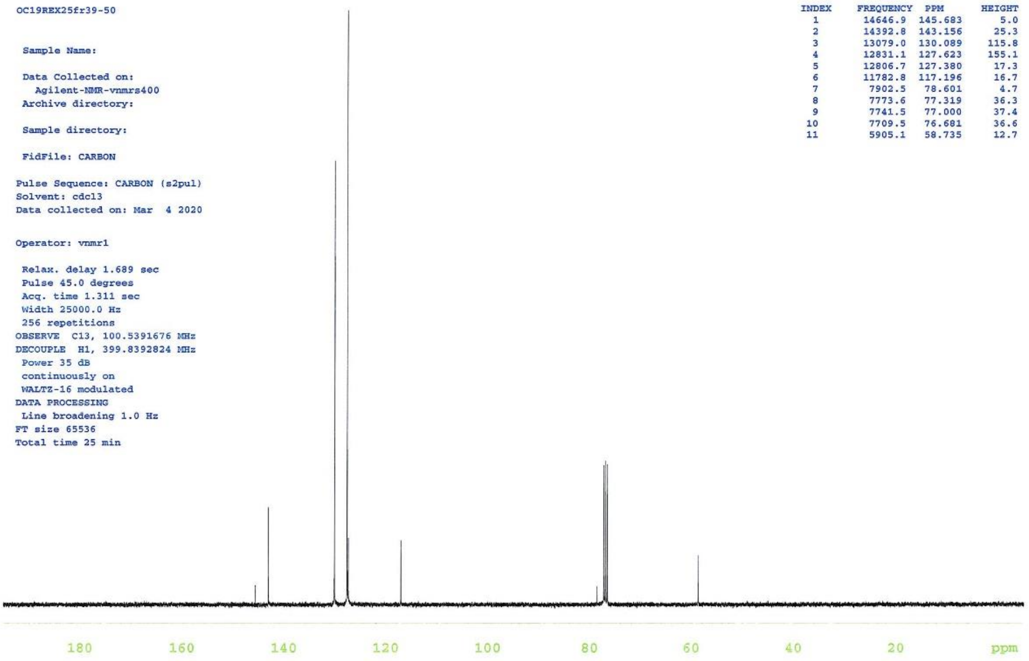

Figure S3. ^{1}H-NMR spectrum of **4d** (400 MHz, $CDCl_3$)

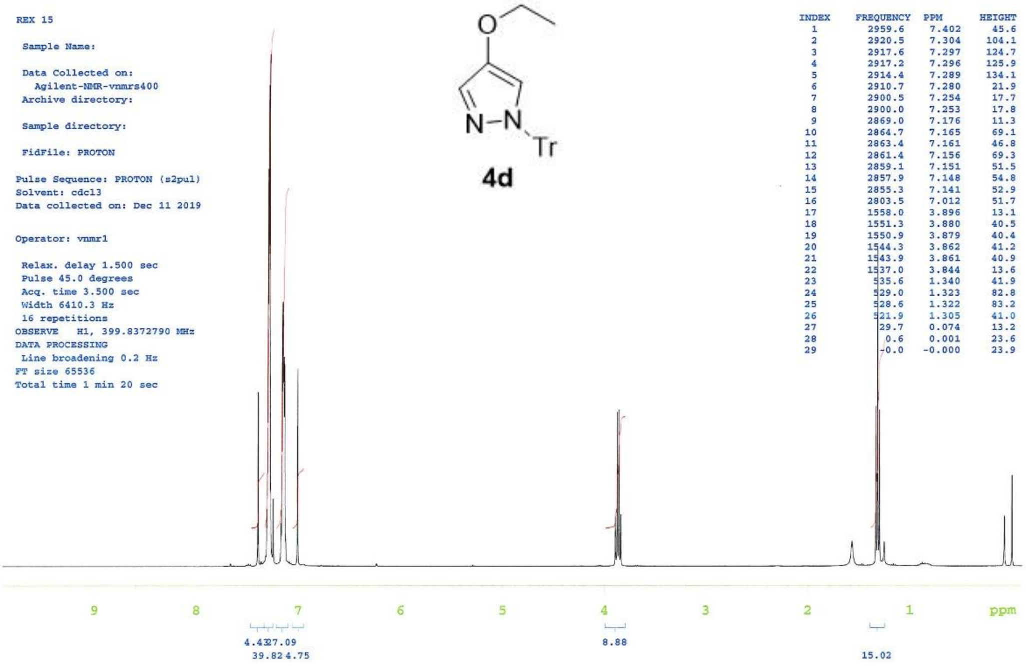

Figure S4. ^{13}C-NMR spectrum of **4d** (100 MHz, $CDCl_3$)

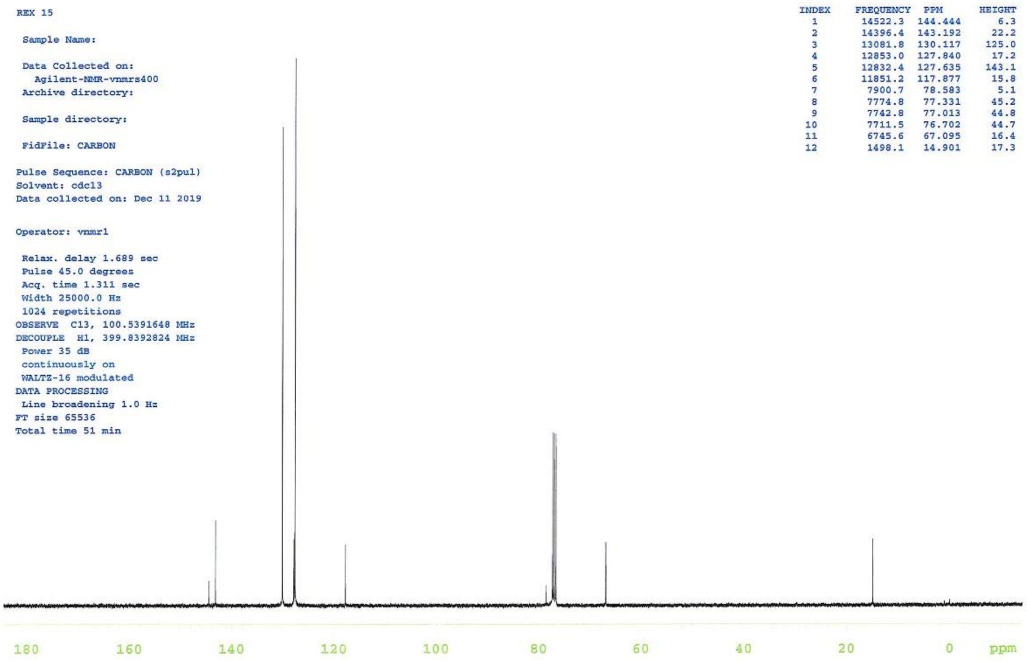

Figure S5. ^{1}H-NMR spectrum of **4e** (400 MHz, $CDCl_3$)

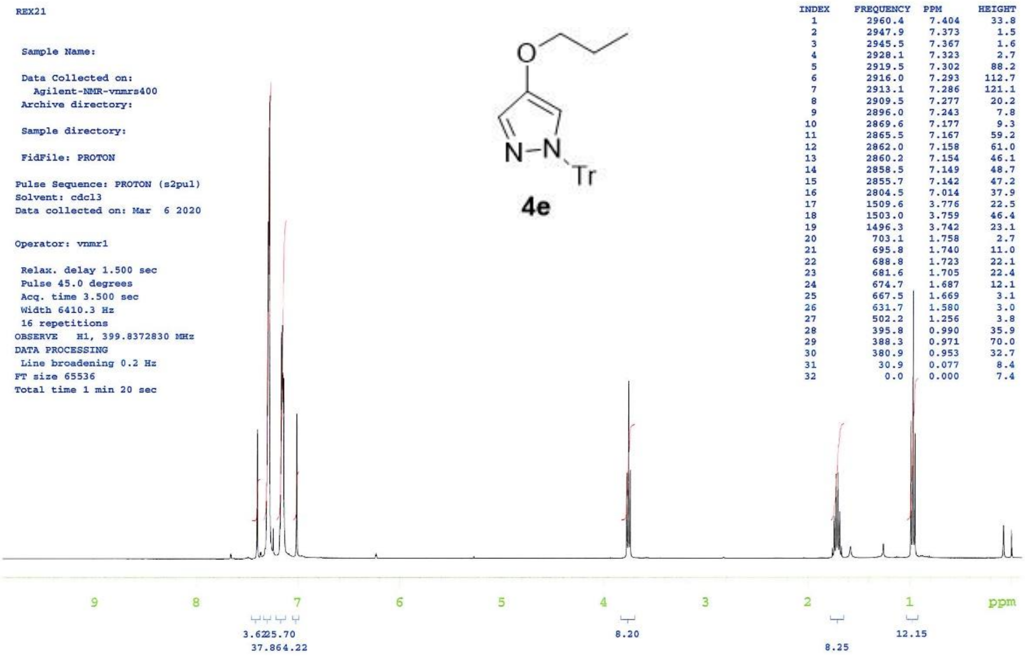

Figure S6. ^{13}C-NMR spectrum of **4e** (100 MHz, $CDCl_3$)

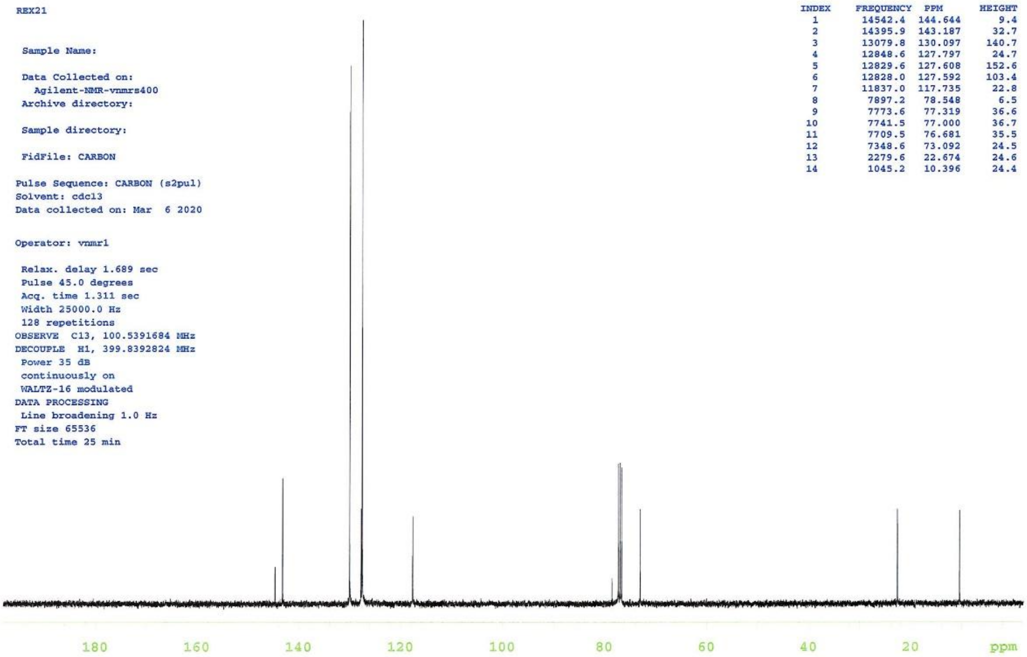

Figure S7. ^{1}H-NMR spectrum of **4f** (400 MHz, $CDCl_3$)

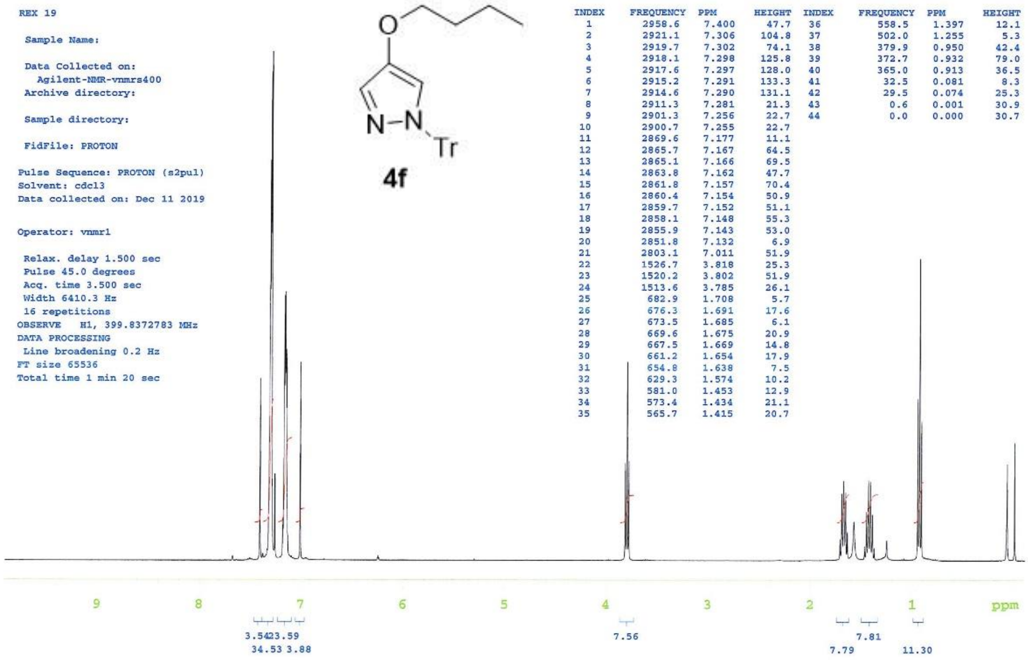

Figure S8. ^{13}C-NMR spectrum of **4f** (100 MHz, $CDCl_3$)

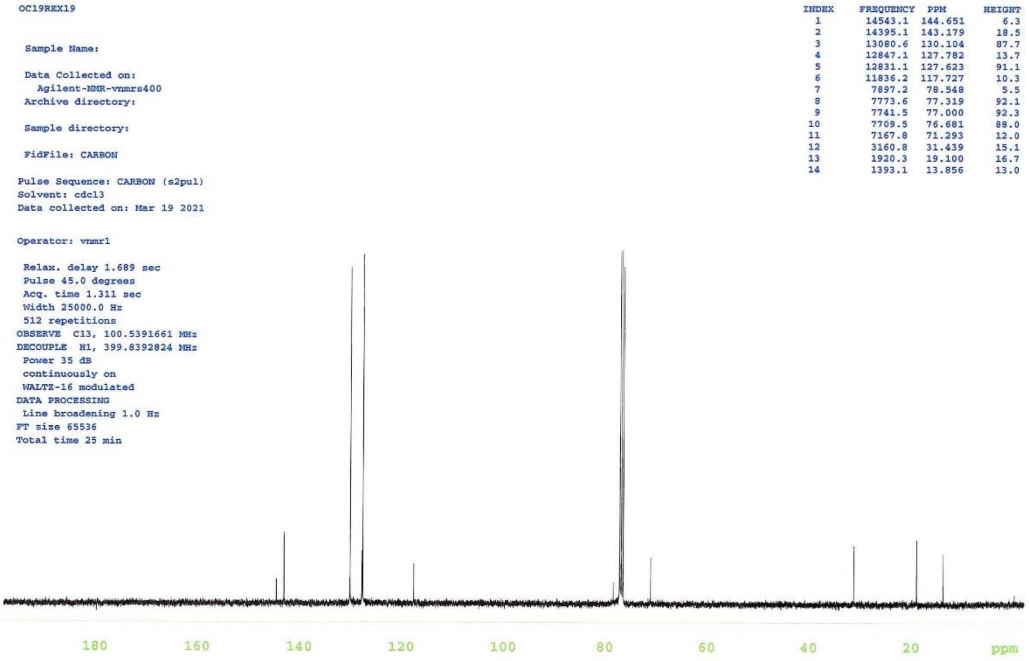

Figure S9. ^{1}H-NMR spectrum of **4g** (400 MHz, $CDCl_3$)

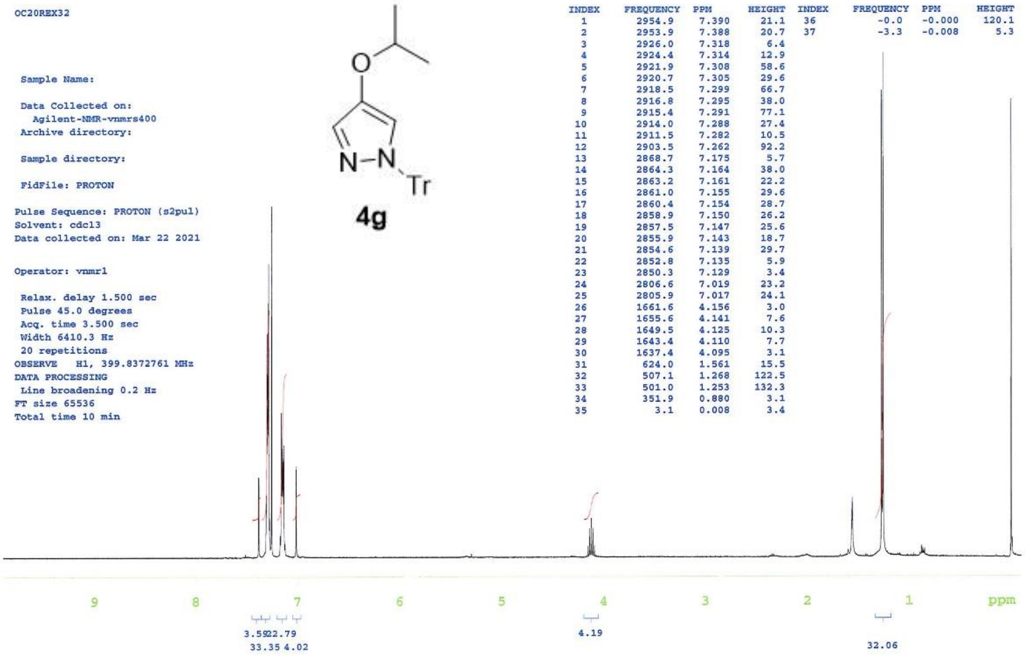

Figure S10. ^{13}C-NMR spectrum of **4g** (100 MHz, $CDCl_3$)

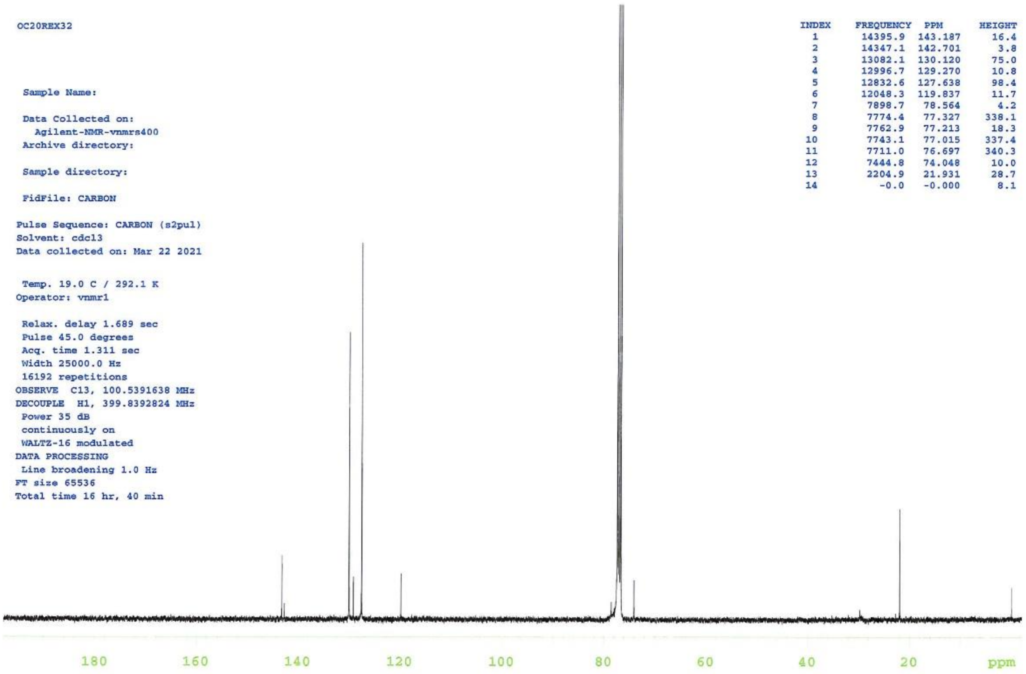

Figure S11. ^{1}H-NMR spectrum of **4i** (400 MHz, $CDCl_3$)

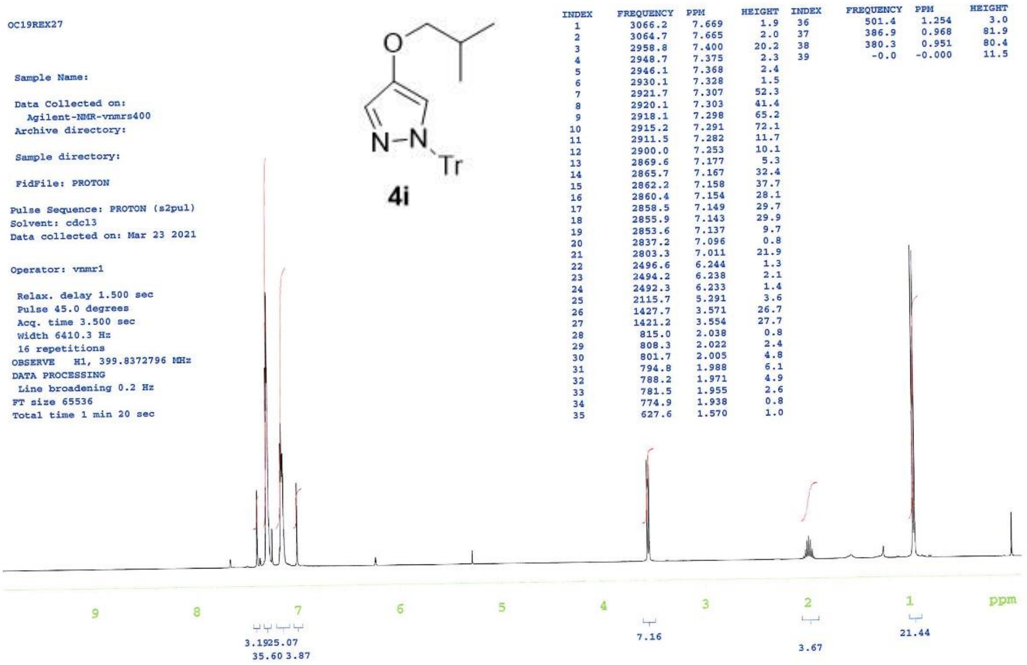

Figure S12. ^{13}C-NMR spectrum of **4i** (100 MHz, $CDCl_3$)

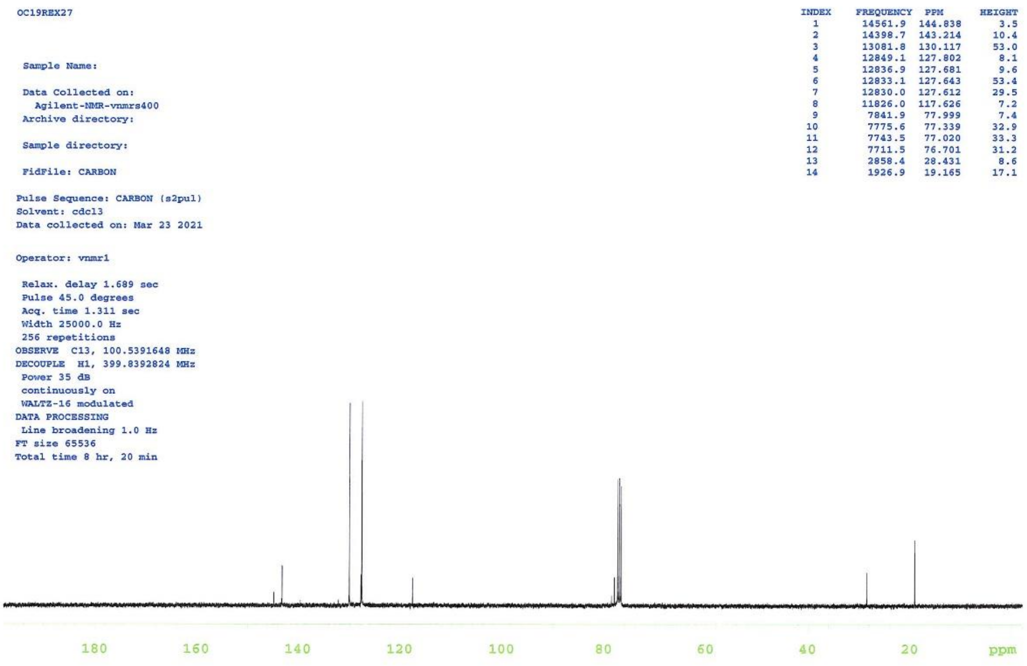

Figure S13. ^{1}H-NMR spectrum of **4k** (400 MHz, $CDCl_3$)

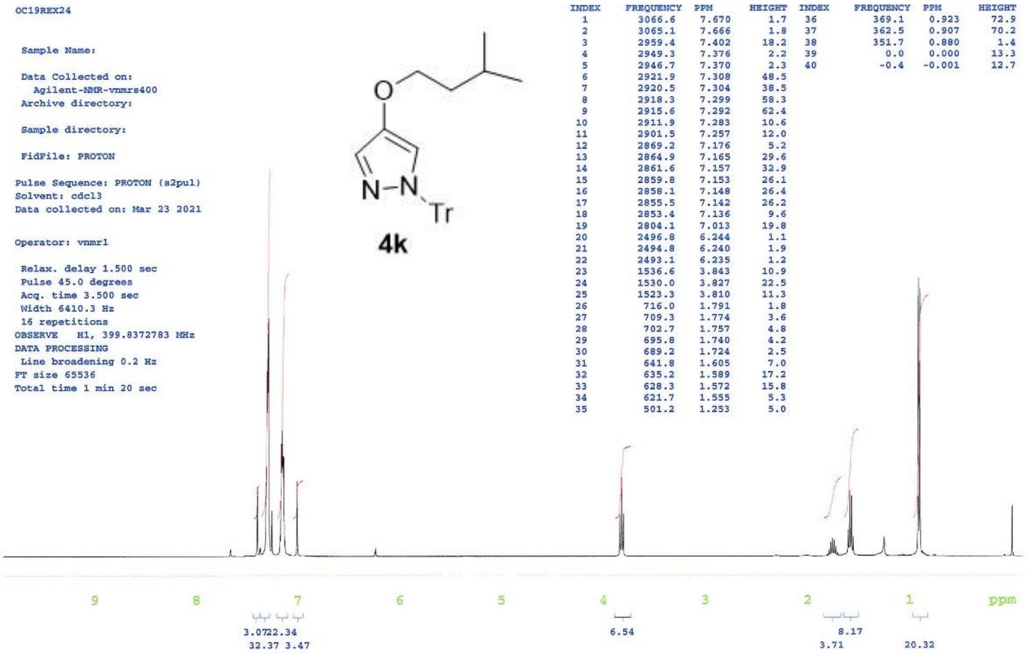

Figure S14. ^{13}C-NMR spectrum of **4k** (100 MHz, $CDCl_3$)

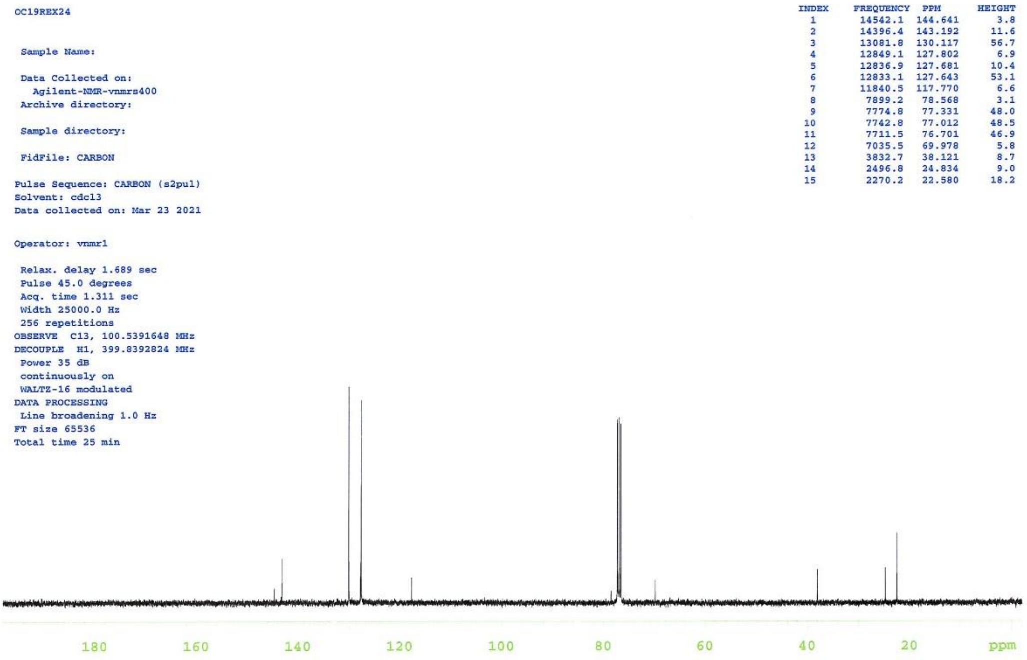

Figure S15. ^{1}H-NMR spectrum of **4l** (400 MHz, $CDCl_3$)

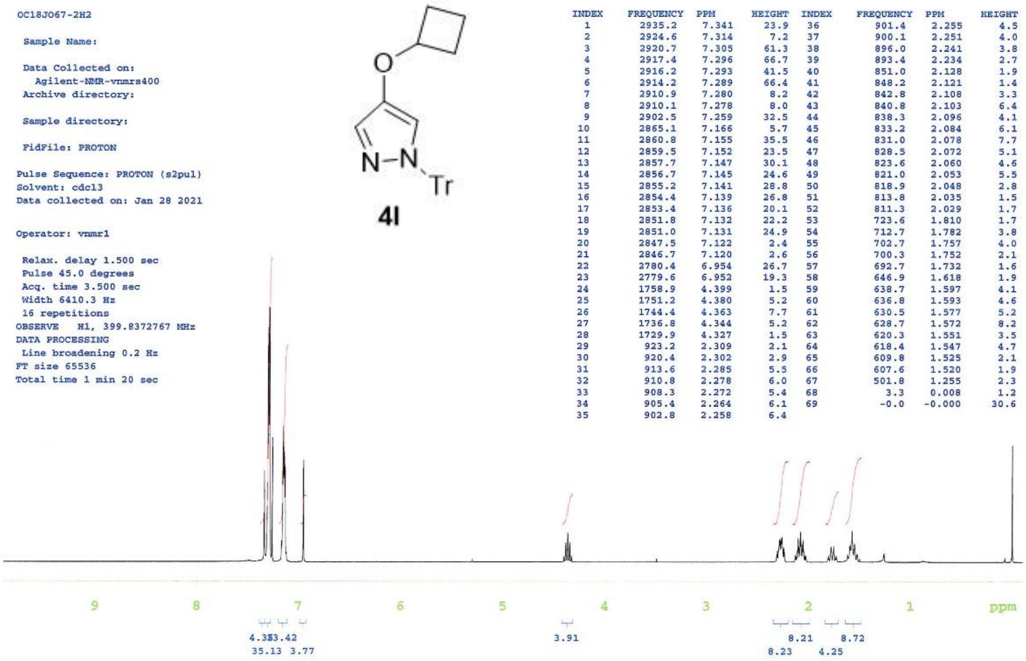

INDEX	FREQUENCY	PPM	HEIGHT	INDEX	FREQUENCY	PPM	HEIGHT
1	2935.2	7.341	23.9	36	901.4	2.255	4.5
2	2924.6	7.314	7.2	37	900.1	2.251	4.0
3	2920.7	7.305	61.3	38	896.0	2.241	3.8
4	2917.4	7.296	66.7	39	893.4	2.234	2.7
5	2916.2	7.293	41.5	40	851.0	2.128	1.9
6	2914.2	7.289	66.4	41	848.2	2.121	1.4
7	2910.9	7.280	8.2	42	842.8	2.108	3.3
8	2910.1	7.278	8.0	43	840.8	2.103	6.4
9	2902.5	7.259	32.5	44	838.3	2.096	4.1
10	2865.1	7.166	5.7	45	833.2	2.084	6.1
11	2860.8	7.155	35.5	46	831.0	2.078	7.7
12	2859.5	7.152	23.5	47	828.5	2.072	5.1
13	2857.7	7.147	30.1	48	823.6	2.060	4.6
14	2856.7	7.145	24.6	49	821.0	2.053	5.5
15	2855.2	7.141	28.8	50	818.9	2.048	2.8
16	2854.4	7.139	26.8	51	813.8	2.035	1.5
17	2853.4	7.136	20.1	52	811.3	2.029	1.7
18	2851.8	7.132	22.2	53	723.6	1.810	1.7
19	2851.0	7.131	24.9	54	712.7	1.782	3.8
20	2847.5	7.122	2.4	55	702.7	1.757	4.0
21	2846.7	7.120	2.6	56	700.3	1.752	2.1
22	2780.4	6.954	26.7	57	692.7	1.732	1.6
23	2779.6	6.952	19.3	58	646.9	1.618	1.9
24	1758.9	4.399	1.5	59	638.7	1.597	4.1
25	1751.2	4.380	5.2	60	636.8	1.593	4.6
26	1744.4	4.363	7.7	61	630.5	1.577	5.2
27	1736.8	4.344	5.2	62	628.7	1.572	8.2
28	1729.9	4.327	1.5	63	620.3	1.551	3.5
29	923.2	2.309	2.1	64	618.4	1.547	4.7
30	920.4	2.302	2.9	65	609.8	1.525	2.1
31	913.6	2.285	5.5	66	607.6	1.520	1.9
32	910.8	2.278	6.0	67	501.8	1.255	2.3
33	908.3	2.272	5.4	68	3.3	0.008	1.2
34	905.4	2.264	6.1	69	-0.0	-0.000	30.6
35	902.8	2.258	6.4				

Figure S16. ^{13}C-NMR spectrum of **4l** (400 MHz, $CDCl_3$)

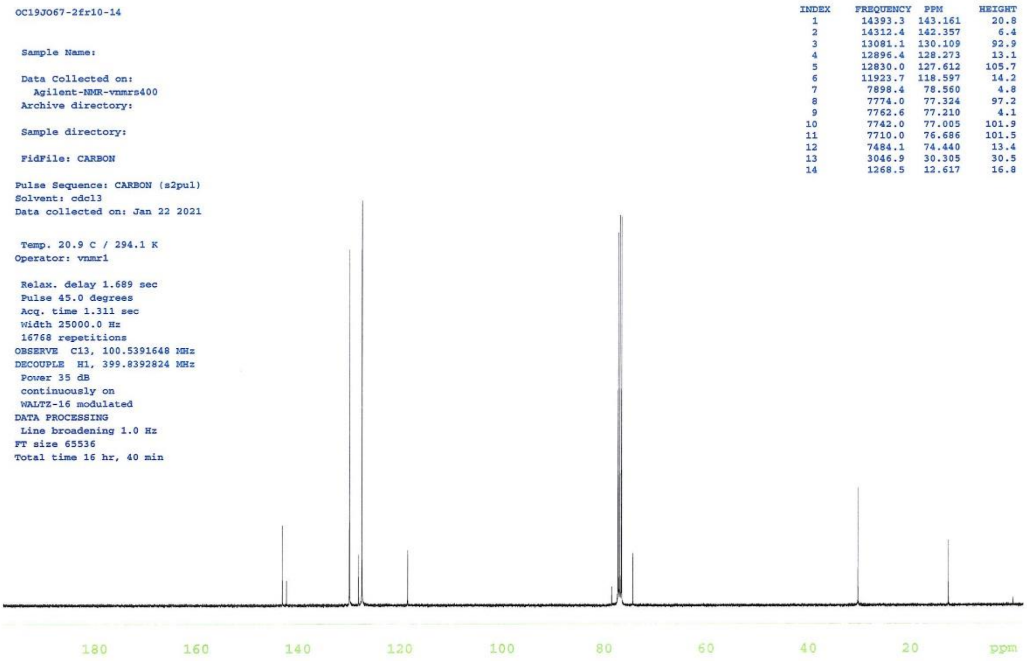

INDEX	FREQUENCY	PPM	HEIGHT
1	14393.3	143.161	20.8
2	14312.4	142.357	6.4
3	13081.1	130.109	92.9
4	12896.4	128.273	13.1
5	12830.0	127.612	105.7
6	11923.7	118.597	14.2
7	7898.4	78.560	4.8
8	7774.0	77.324	97.2
9	7762.6	77.210	4.1
10	7742.0	77.005	101.9
11	7710.0	76.686	101.5
12	7484.1	74.440	13.4
13	3046.9	30.305	30.5
14	1268.5	12.617	16.8

Figure S17. ^{1}H-NMR spectrum of **4m** (400 MHz, $CDCl_3$)

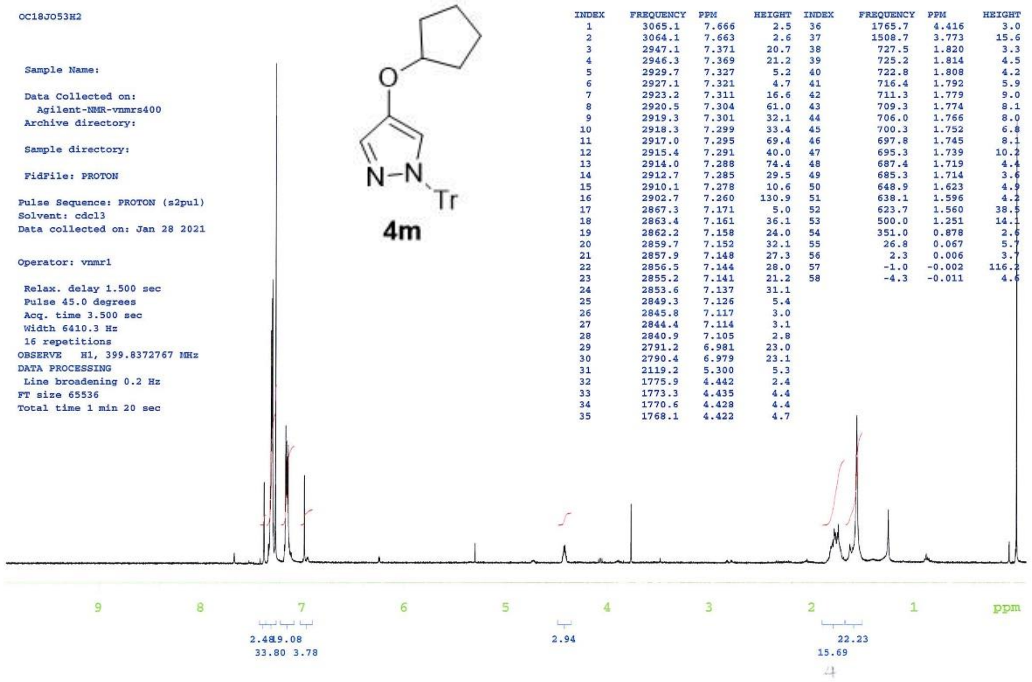

Figure S18. ^{13}C-NMR spectrum of **4m** (100 MHz, $CDCl_3$)

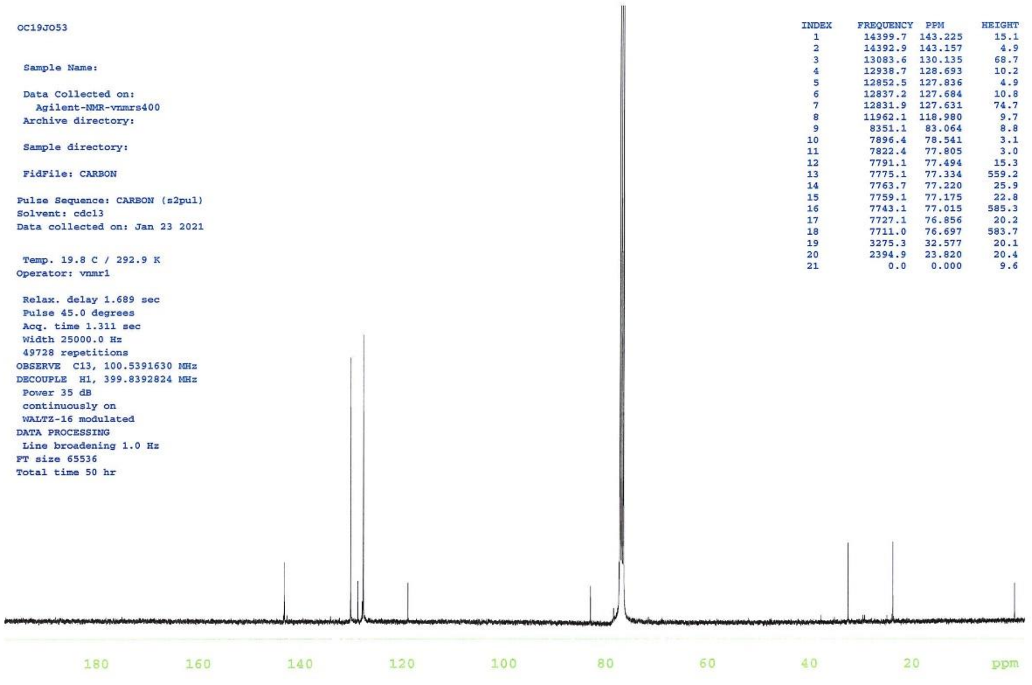

Figure S19. ^{1}H-NMR spectrum of **4n** (400 MHz, $CDCl_3$)

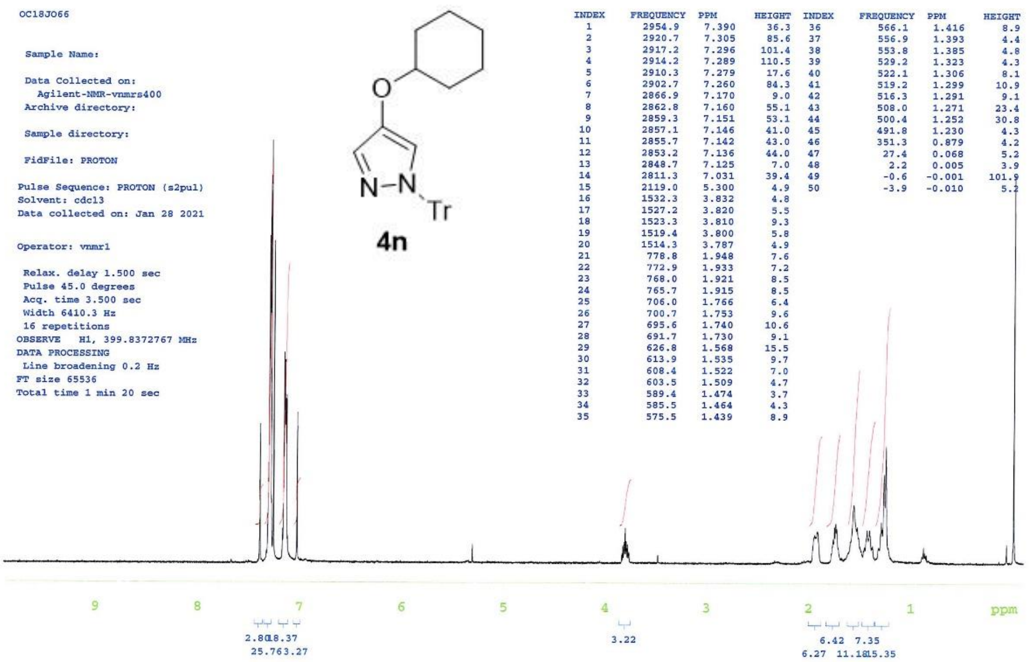

Figure S20. ^{13}C-NMR spectrum of **4n** (400 MHz, $CDCl_3$)

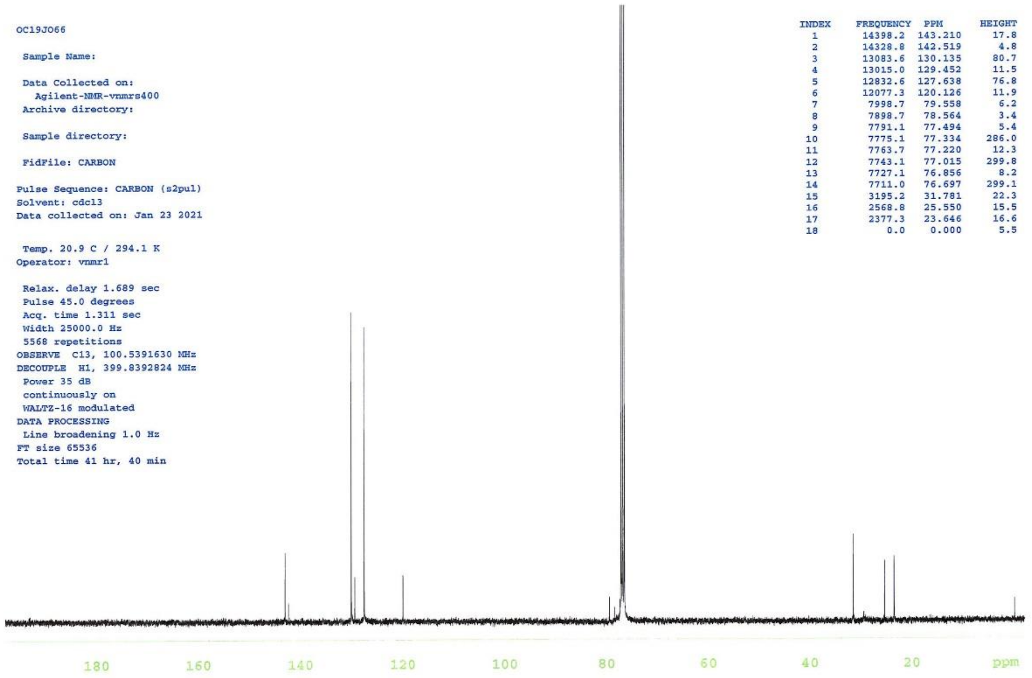

Figure S21. ^{1}H-NMR spectrum of **4o** (400 MHz, $CDCl_3$)

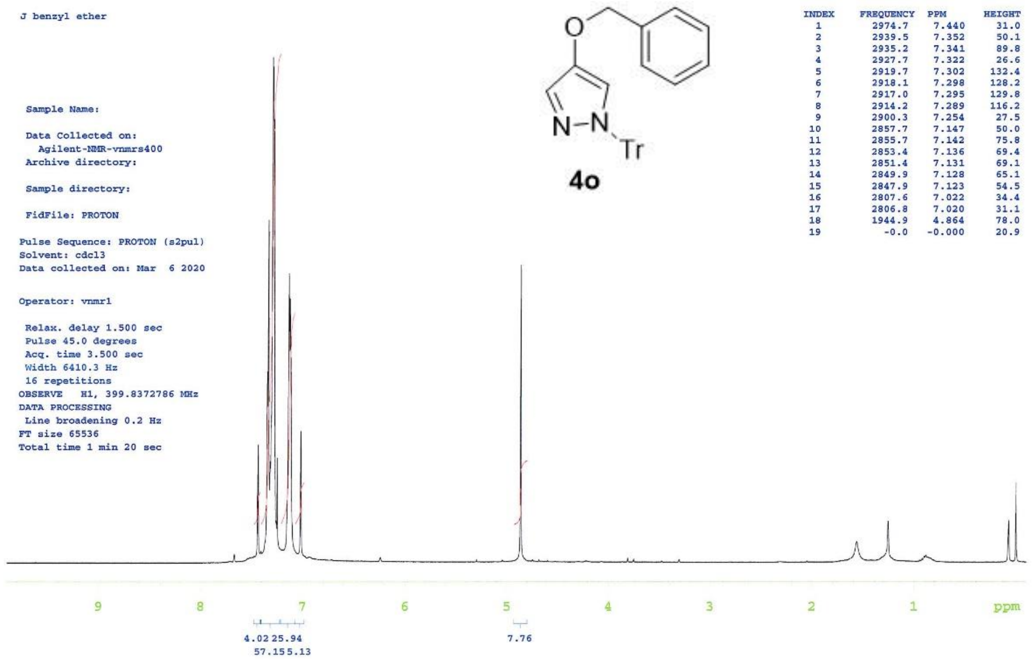

Figure S22. ^{13}C-NMR spectrum of **4o** (100 MHz, $CDCl_3$)

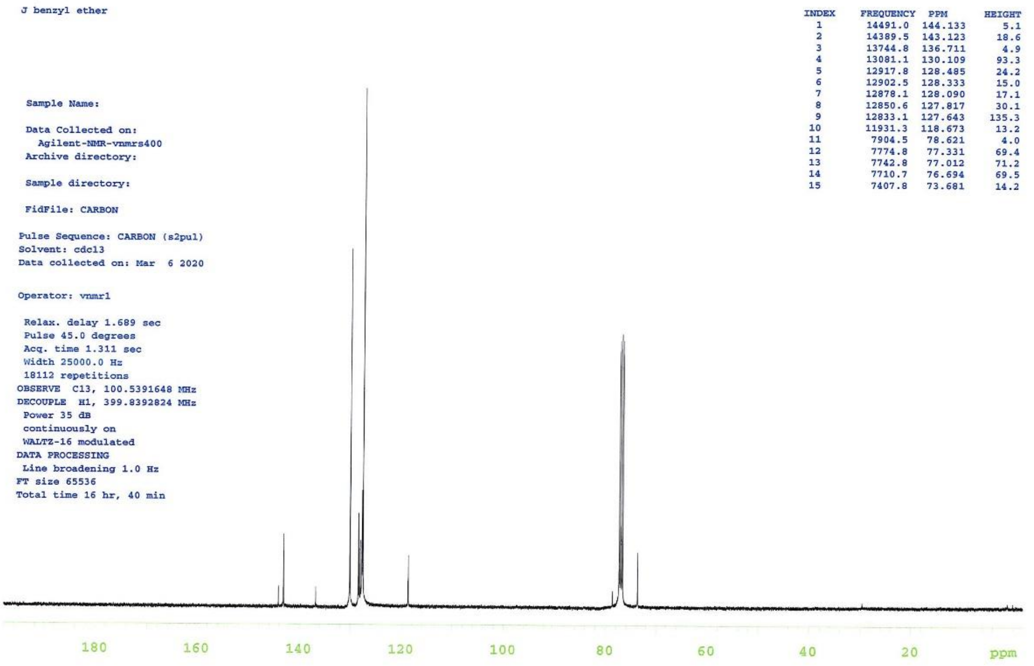

Figure S23. ^{1}H-NMR spectrum of **4r** (400 MHz, $CDCl_3$)

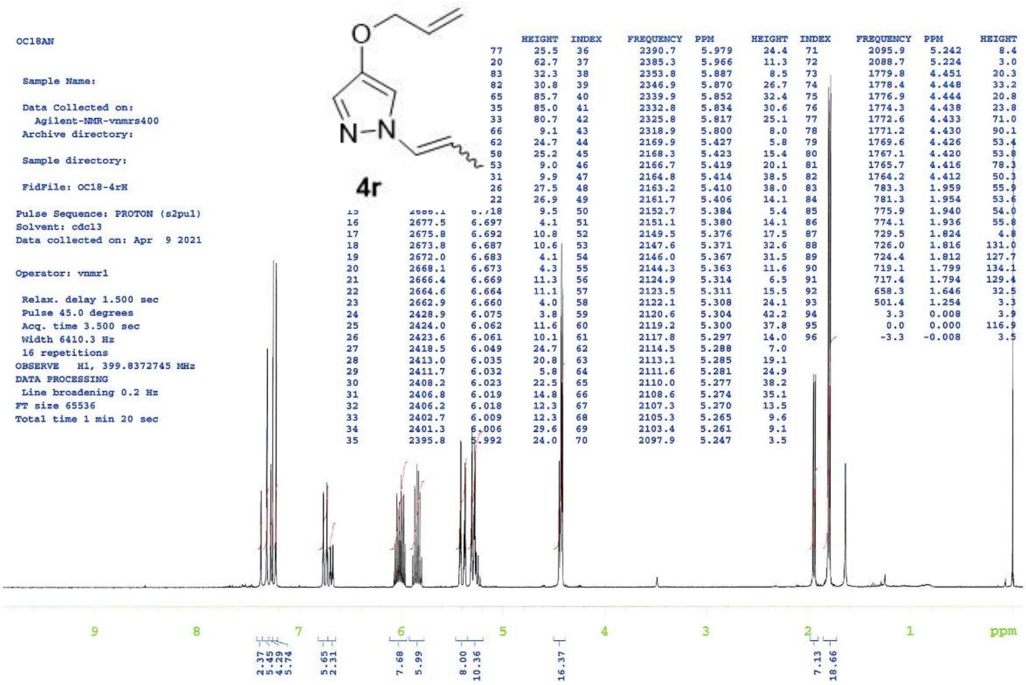

Figure S24. ^{13}C-NMR spectrum of **4r** (100 MHz, $CDCl_3$)

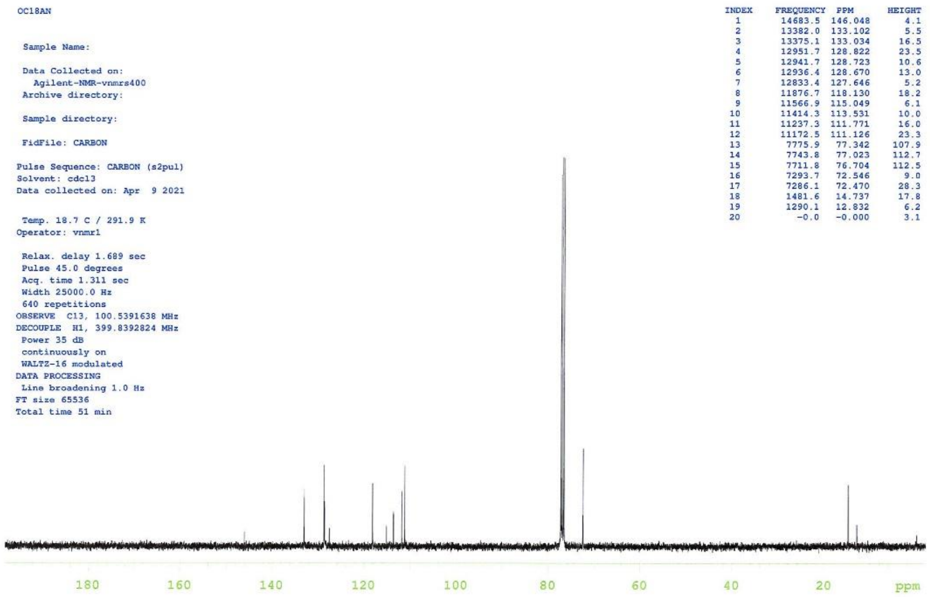

Figure S25. ^{1}H-NMR spectrum of **4t** (400 MHz, $CDCl_3$)

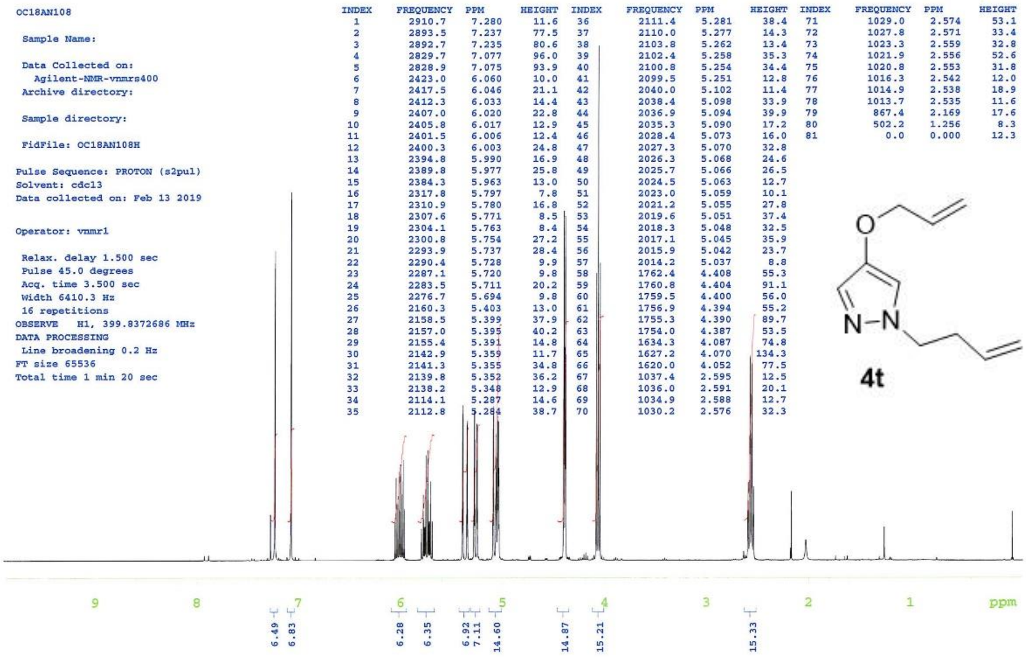

Figure S26. ^{13}C-NMR spectrum of **4t** (100 MHz, $CDCl_3$)

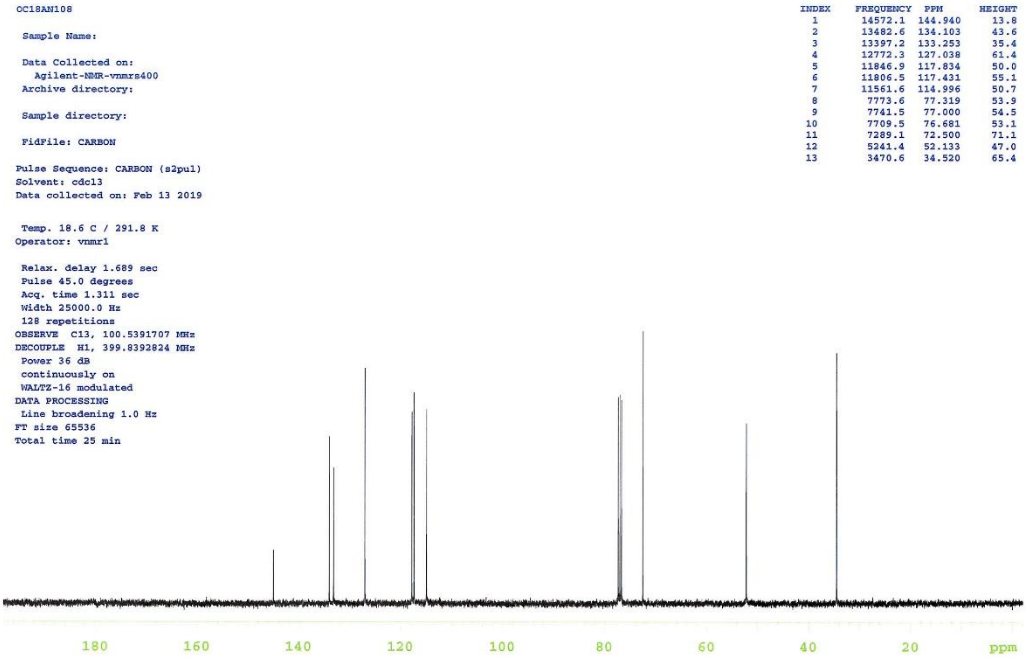

Figure S27. ^{1}H-NMR spectrum of **2c** (400 MHz, $CDCl_3$)

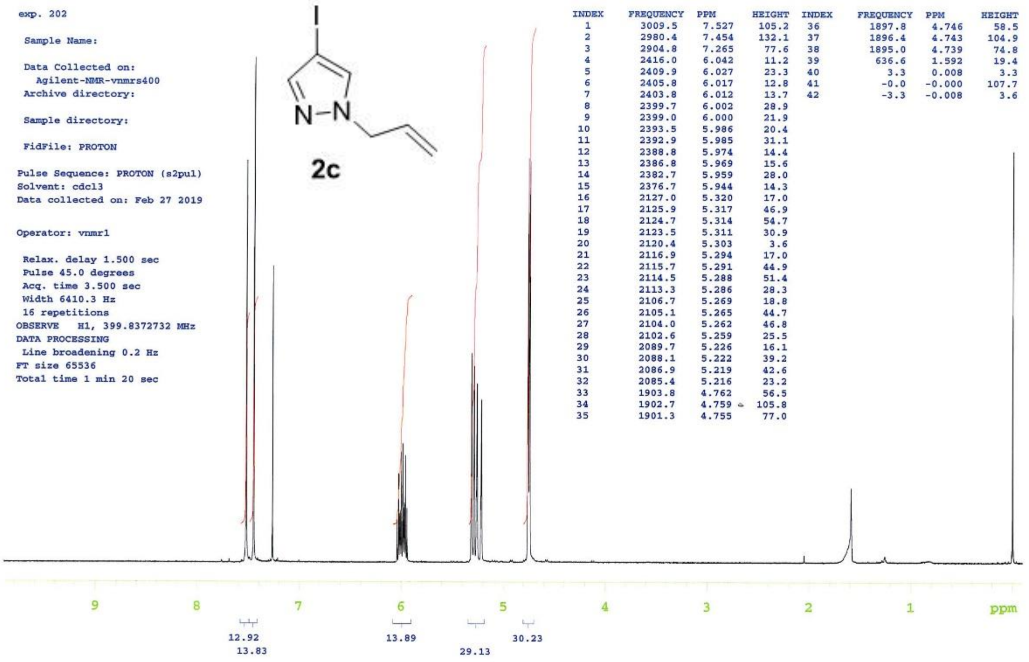

Figure S28. ^{13}C-NMR spectrum of **2c** (400 MHz, $CDCl_3$)

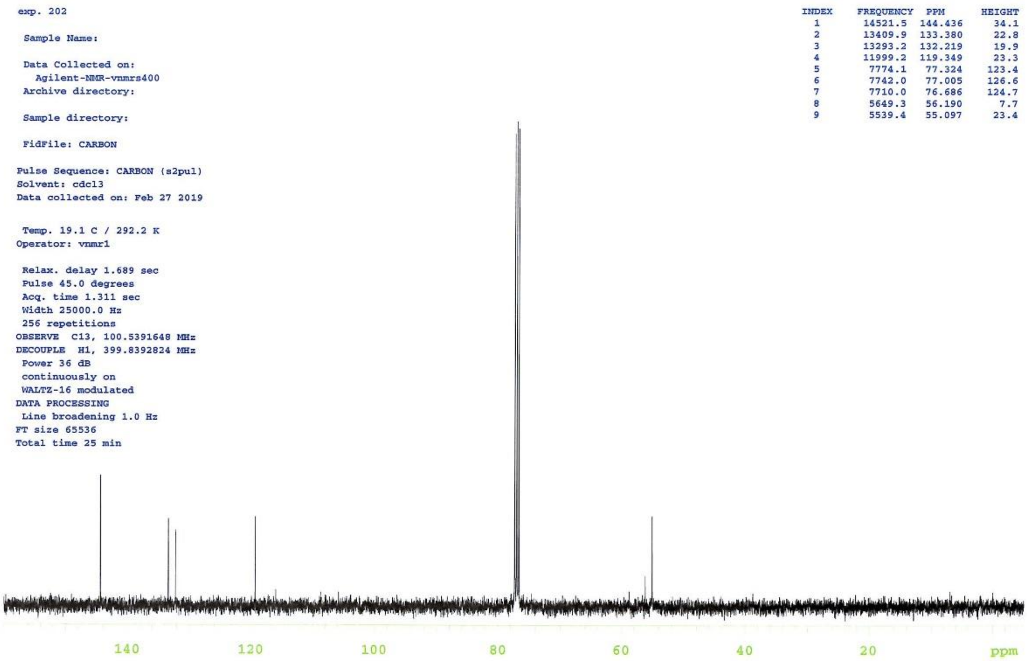

Figure S29. ^{1}H-NMR spectrum of **12** (400 MHz, $CDCl_3$)

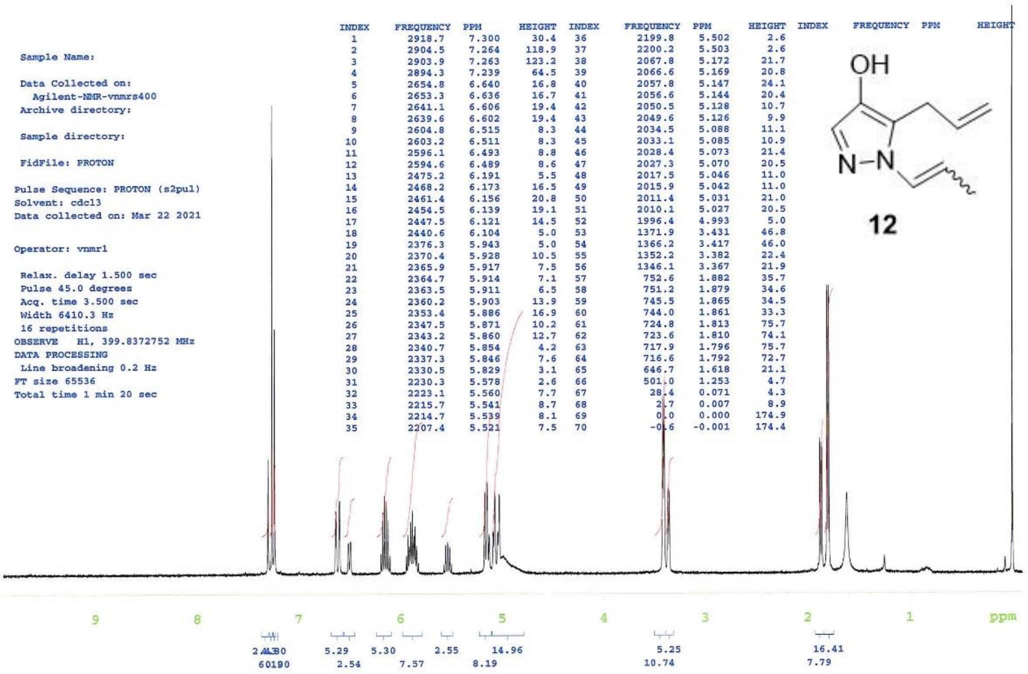

Figure S30. ^{13}C-NMR spectrum of **12** (100 MHz, $CDCl_3$)

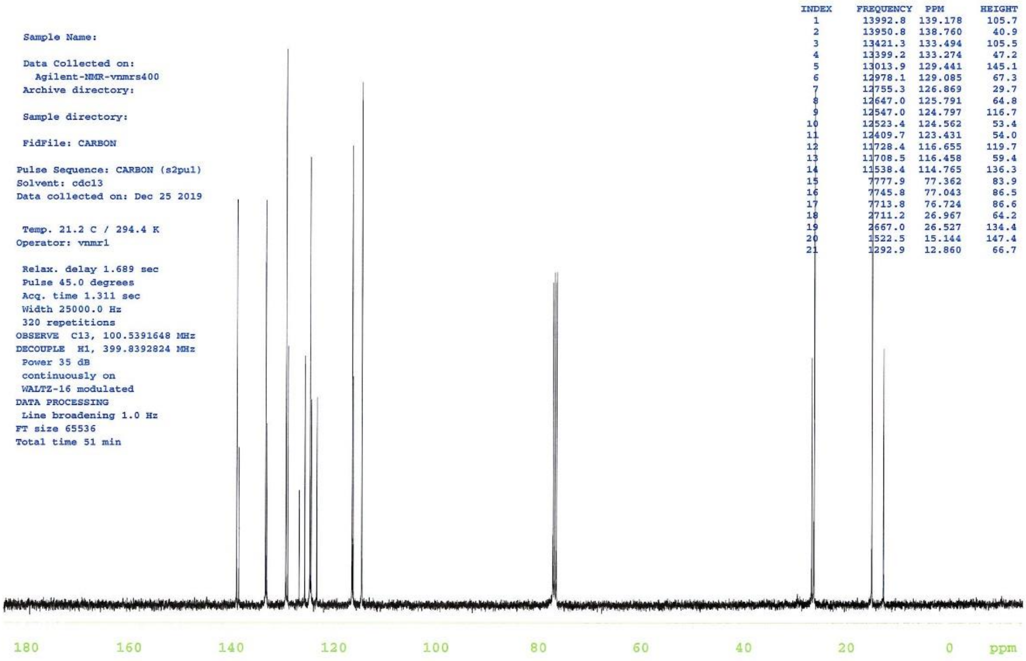

Figure S31. ^{1}H-NMR spectrum of ***(Z)*-13** (600 MHz, $CDCl_3$)

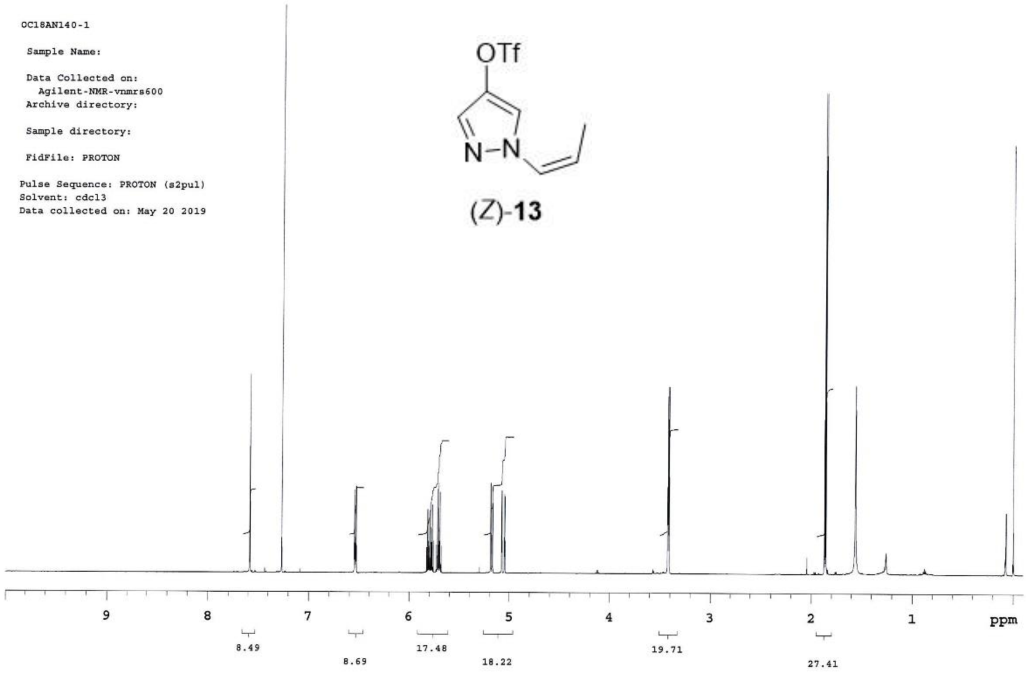

OC18AN140-1

INDEX	FREQUENCY	PPM	HEIGHT	INDEX	FREQUENCY	PPM	HEIGHT
1	4548.9	7.583	737.7	51	2047.0	3.412	695.1
2	4356.1	7.261	2840.3	52	2045.3	3.409	361.6
3	3930.0	6.551	111.2	53	1227.2	2.046	63.6
4	3928.3	6.548	308.5	54	1126.2	1.877	28.8
5	3926.2	6.545	285.3	55	1123.3	1.872	1787.0
6	3924.4	6.542	105.8	56	1121.5	1.870	1499.7
7	3921.2	6.536	108.8	57	1119.2	1.866	64.7
8	3919.5	6.534	322.5	58	1115.9	1.860	1552.6
9	3917.7	6.531	321.0	59	1114.2	1.857	1784.7
10	3915.9	6.528	102.5	60	935.8	1.560	700.3
11	3494.8	5.826	95.9	61	762.9	1.272	31.7
12	3489.0	5.816	236.7	62	755.6	1.260	76.0
13	3484.9	5.809	118.5	63	753.0	1.255	82.4
14	3483.1	5.806	100.2	64	748.6	1.248	31.3
15	3478.7	5.799	222.2	65	528.2	0.880	25.0
16	3477.8	5.797	128.0	66	48.4	0.081	32.6
17	3472.8	5.789	134.2	67	47.5	0.079	49.4
18	3472.0	5.788	254.7	68	46.7	0.078	49.0
19	3467.9	5.781	114.5	69	45.8	0.076	59.5
20	3465.8	5.777	114.0	70	44.9	0.075	64.2
21	3461.7	5.770	254.1	71	41.7	0.069	230.7
22	3455.8	5.761	119.5	72	3.2	0.005	42.0
23	3439.7	5.734	105.7	73	0.0	0.000	1593.3
24	3432.6	5.722	313.1	74	-3.5	-0.006	37.2
25	3431.2	5.720	105.7				
26	3425.3	5.710	335.8				
27	3423.8	5.707	324.7				
28	3418.3	5.698	117.9				
29	3416.8	5.696	300.8				
30	3409.5	5.683	100.4				
31	3113.4	5.190	126.3				
32	3111.6	5.187	334.2				
33	3110.4	5.185	336.3				
34	3109.0	5.183	147.3				
35	3103.1	5.173	140.6				
36	3101.6	5.170	311.6				
37	3100.5	5.168	319.3				
38	3098.7	5.165	121.9				
39	3046.2	5.078	140.1				
40	3044.4	5.075	308.1				
41	3043.2	5.073	289.5				
42	3041.5	5.070	122.9				
43	3029.2	5.049	133.2				
44	3027.4	5.047	294.4				
45	3026.2	5.045	284.7				
46	3024.5	5.042	120.5				
47	2054.7	3.425	374.6				
48	2052.9	3.422	694.8				
49	2051.4	3.420	383.4				
50	2048.5	3.415	384.8				

Figure S32. ^{13}C-NMR spectrum of (*Z*)**-13** (150 MHz, $CDCl_3$)

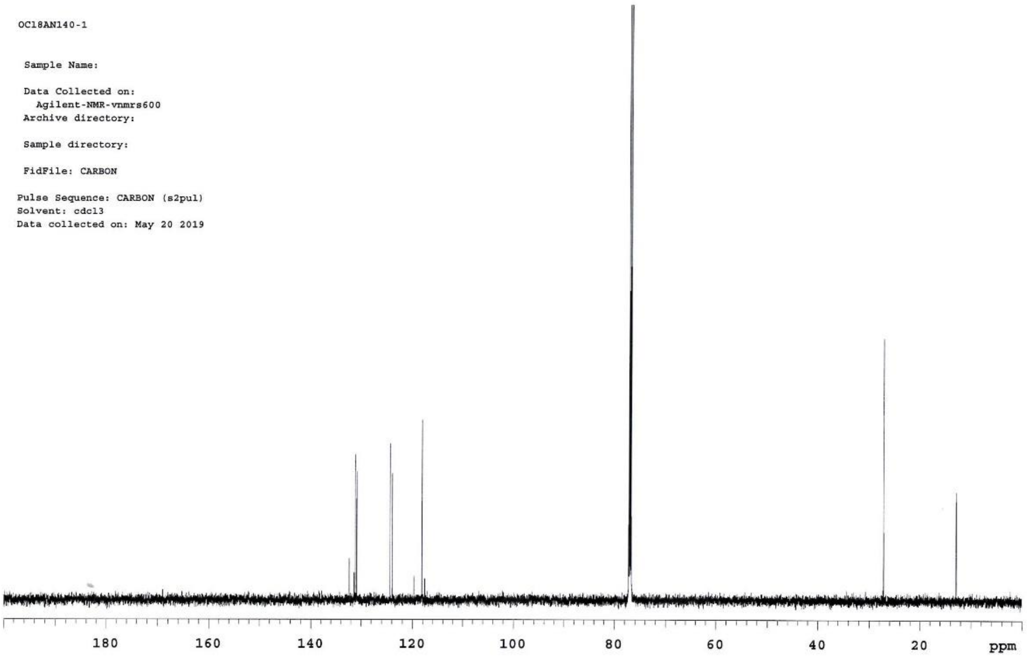

OC18AN140-1

INDEX	FREQUENCY	PPM	HEIGHT
1	19968.0	132.375	10.8
2	19818.9	131.387	7.1
3	19785.4	131.165	38.1
4	19740.3	130.866	33.5
5	18757.7	124.352	40.9
6	18694.1	123.930	33.1
7	18060.7	119.731	6.2
8	17823.7	118.160	47.1
9	17739.3	117.600	5.5
10	11647.3	77.215	519.8
11	11615.0	77.000	520.4
12	11583.8	76.793	482.4
13	4102.3	27.196	68.5
14	1944.1	12.888	28.5

Figure S33. ^{1}H-NMR spectrum of (*E*)-**13** (600 MHz, $CDCl_3$)

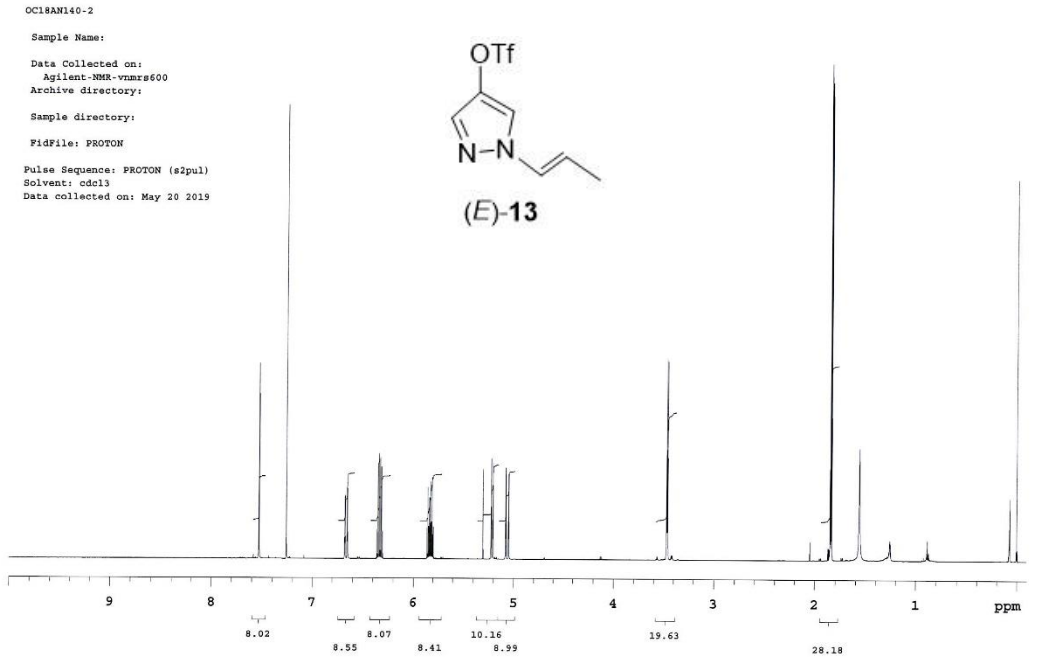

OC18AN140-2

INDEX	FREQUENCY	PPM	HEIGHT	INDEX	FREQUENCY	PPM	HEIGHT
1	4517.5	7.530	801.4	51	3044.1	5.074	144.4
2	4356.1	7.261	1856.8	52	3031.5	5.053	155.4
3	4005.1	6.676	86.9	53	3029.7	5.050	348.4
4	4004.0	6.674	251.7	54	3028.6	5.049	332.0
5	4003.4	6.673	254.0	55	3028.0	5.048	177.4
6	4002.2	6.672	260.9	56	3026.8	5.046	151.6
7	4001.6	6.671	252.1	57	2082.8	3.472	429.6
8	4000.4	6.669	92.8	58	2081.1	3.469	821.8
9	3999.9	6.668	80.8	59	2079.3	3.466	445.6
10	3991.9	6.654	93.4	60	2077.0	3.462	448.9
11	3991.3	6.653	100.9	61	2075.2	3.459	811.3
12	3990.2	6.651	286.1	62	2073.4	3.456	417.6
13	3989.6	6.650	279.2	63	1227.2	2.046	77.4
14	3988.4	6.649	288.2	64	1123.3	1.872	49.1
15	3987.8	6.648	267.8	65	1121.5	1.870	50.7
16	3986.7	6.646	101.5	66	1116.2	1.861	51.1
17	3816.8	6.362	121.4	67	1114.5	1.858	44.8
18	3809.7	6.351	393.7	68	1112.7	1.855	29.8
19	3803.0	6.339	431.6	69	1109.5	1.849	2028.8
20	3796.2	6.328	417.0	70	1107.7	1.847	1976.9
21	3789.2	6.316	378.0	71	1105.7	1.843	69.9
22	3782.1	6.305	114.3	72	1102.4	1.838	1952.9
23	3516.6	5.862	128.4	73	1100.7	1.835	1898.7
24	3510.7	5.852	292.9	74	1096.9	1.828	29.5
25	3506.3	5.845	136.3	75	937.5	1.563	458.1
26	3504.8	5.842	120.1	76	762.9	1.272	40.1
27	3500.7	5.836	253.9	77	760.6	1.268	28.5
28	3499.5	5.834	135.1	78	758.0	1.263	31.9
29	3494.8	5.826	148.5	79	755.6	1.260	84.6
30	3493.7	5.824	317.1	80	753.0	1.255	85.1
31	3489.3	5.816	153.8	81	748.6	1.248	35.5
32	3487.8	5.814	152.7	82	537.3	0.896	33.0
33	3483.4	5.807	336.8	83	530.2	0.884	86.2
34	3477.5	5.797	133.2	84	523.2	0.872	36.2
35	3179.7	5.300	368.5	85	48.7	0.081	35.0
36	3134.2	5.225	152.1	86	47.8	0.080	53.5
37	3133.3	5.223	215.7	87	47.0	0.078	54.9
38	3132.7	5.222	387.7	88	46.1	0.077	65.7
39	3131.6	5.220	414.0	89	45.2	0.075	83.5
40	3131.0	5.219	213.4	90	44.6	0.074	84.7
41	3130.1	5.218	157.0	91	43.1	0.072	198.9
42	3124.2	5.208	160.4	92	42.0	0.070	257.0
43	3122.5	5.205	396.3	93	3.2	0.005	41.2
44	3121.6	5.204	334.4	94	0.0	0.000	1555.7
45	3119.8	5.201	167.7	95	-3.2	-0.005	33.0
46	3048.5	5.082	150.7				
47	3047.6	5.080	190.0				
48	3046.8	5.079	375.2				
49	3045.9	5.077	346.2				
50	3045.0	5.076	199.5				

Figure S34. ^{13}C-NMR spectrum of (*E*)-**13** (150 MHz, $CDCl_3$)

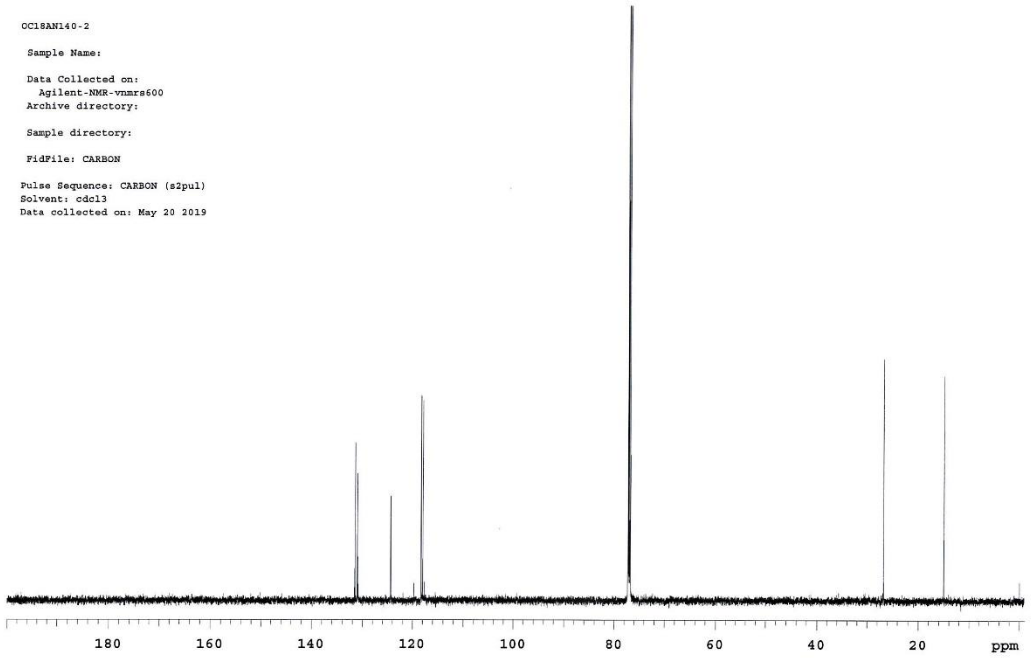

OC18AN140-2

INDEX	FREQUENCY	PPM	HEIGHT
1	19853.6	131.617	8.3
2	19828.1	131.448	41.3
3	19764.6	131.027	33.2
4	19747.2	130.912	11.4
5	18754.2	124.329	27.3
6	18058.3	119.715	4.4
7	17839.9	118.267	53.6
8	17790.2	117.938	52.3
9	17737.0	117.585	4.8
10	11647.3	77.215	394.6
11	11615.0	77.000	398.8
12	11583.8	76.793	362.5
13	4030.7	26.721	63.3
14	2264.3	15.011	58.8
15	-2.5	-0.017	4.8

Figure S35. ^{1}H-NMR spectrum of **14** (400 MHz, $CDCl_3$)

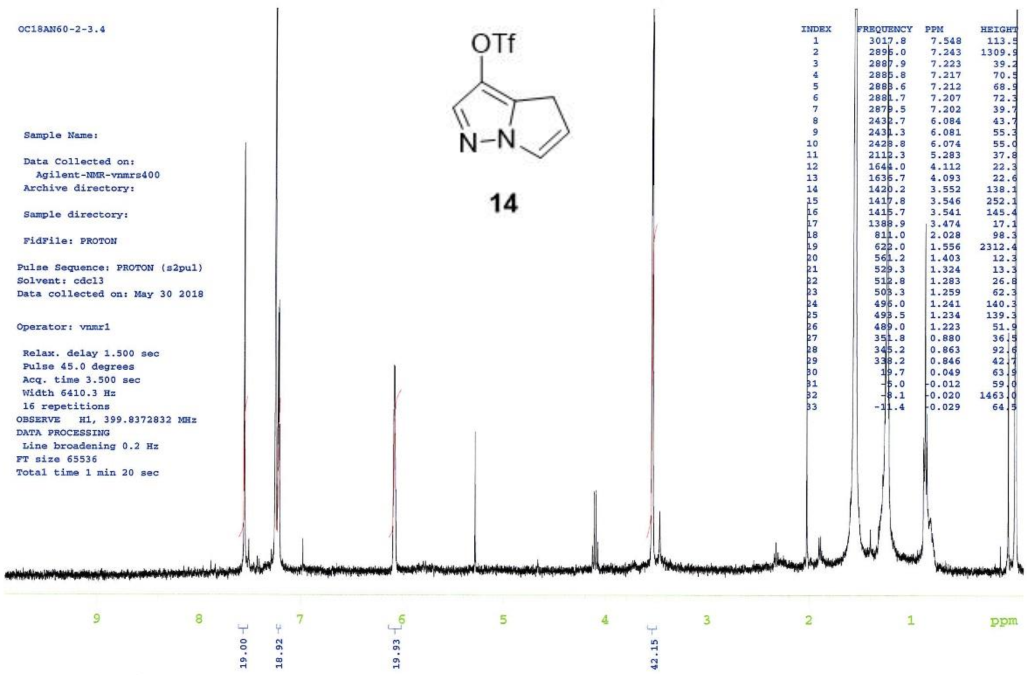

Figure S36. ^{13}C-NMR spectrum of **14** (100 MHz, $CDCl_3$)

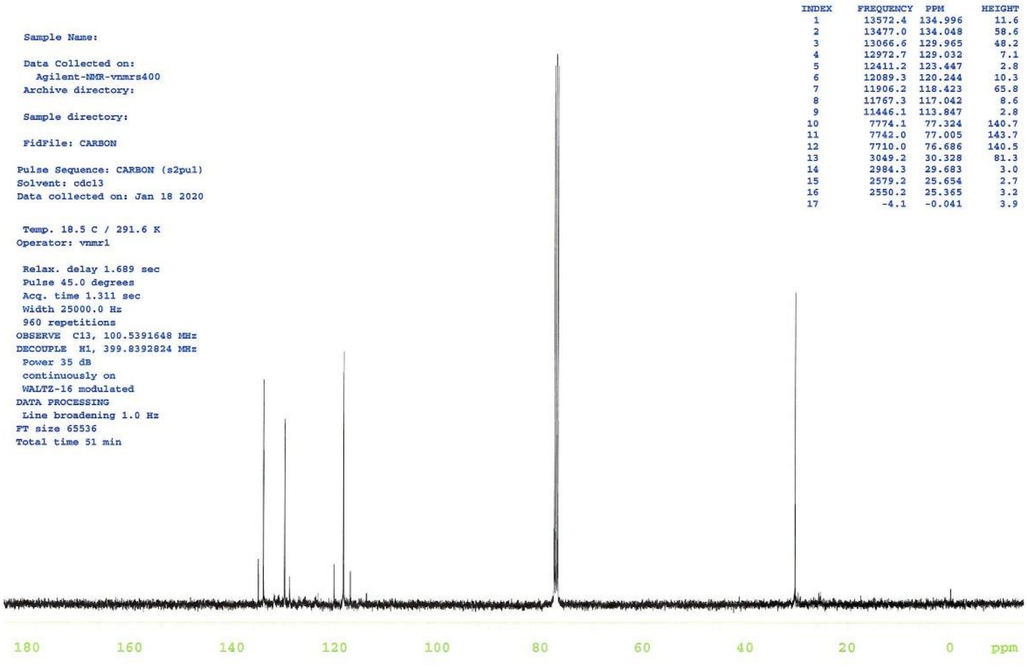

Figure S37. ^{1}H-NMR spectrum of **2d** (400 MHz, $CDCl_3$)

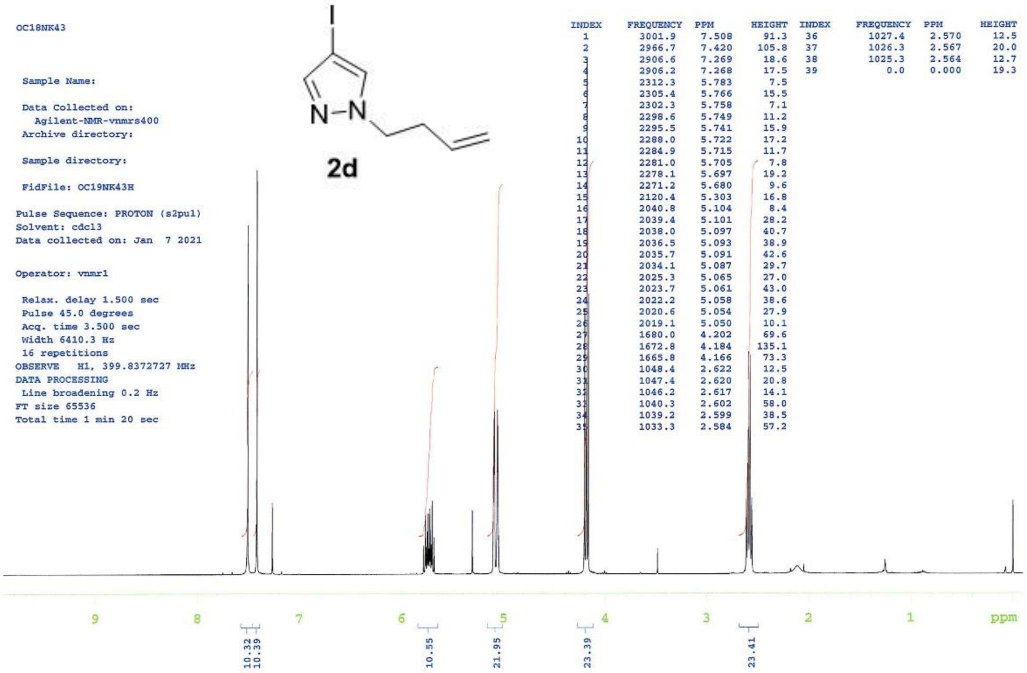

Figure S38. ^{13}C-NMR spectrum of **2d** (100 MHz, $CDCl_3$)

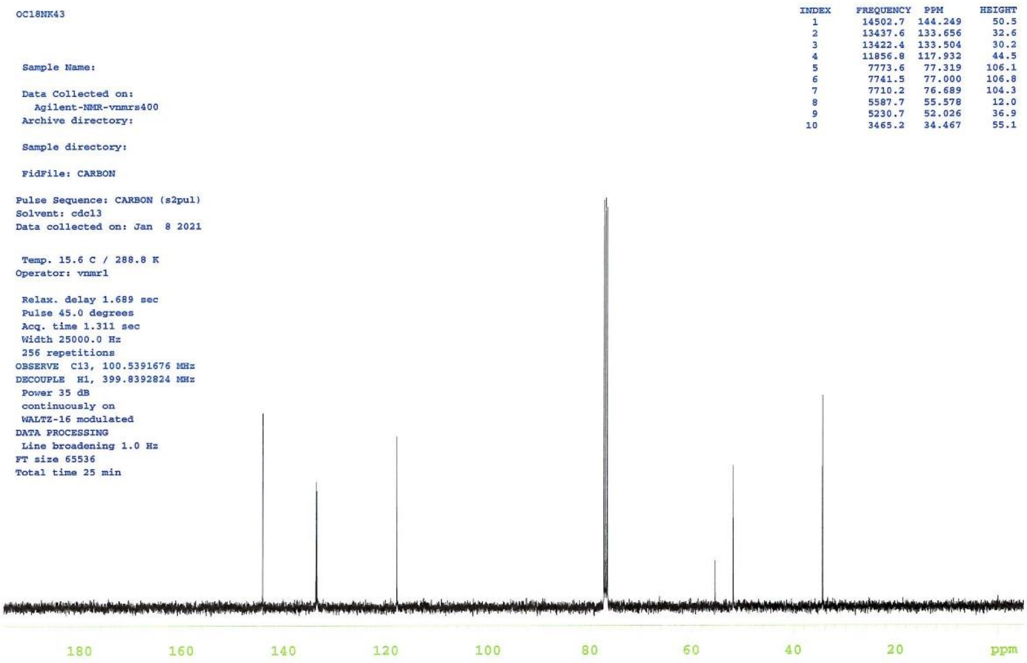

Figure S39. ^{1}H-NMR spectrum of **16** (400 MHz, $CDCl_3$)

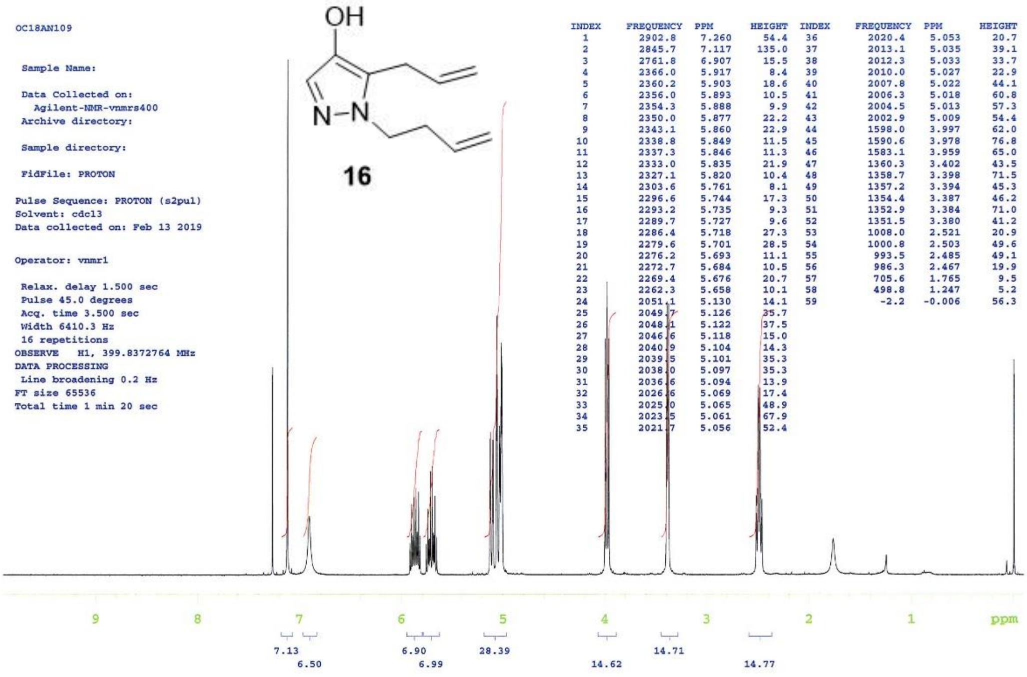

Figure S40. ^{1}H-NMR spectrum of **16** (100 MHz, $CDCl_3$)

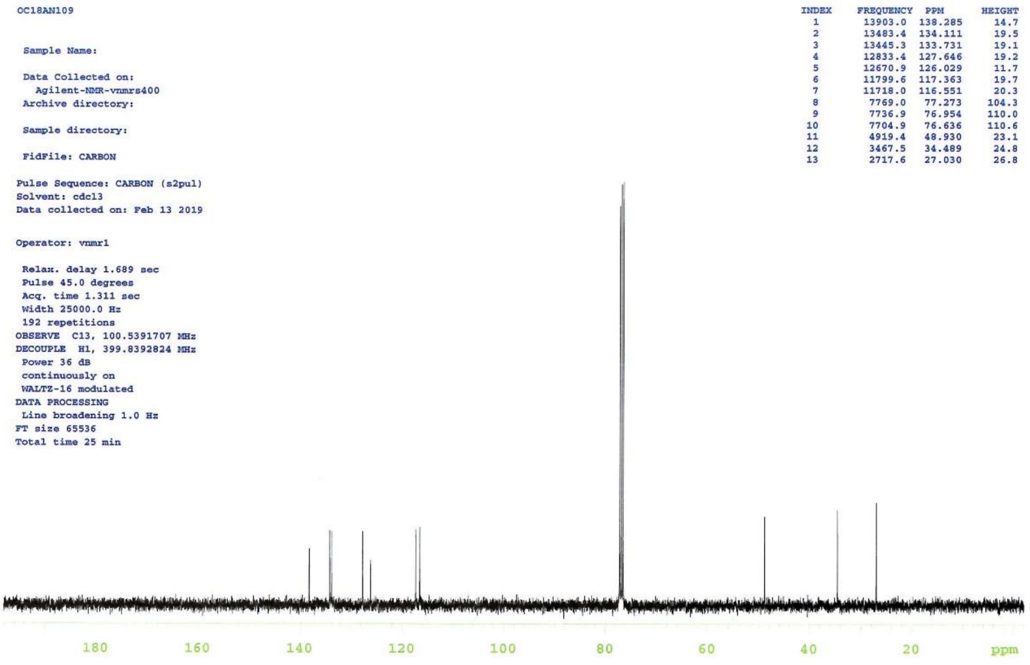

Figure S41. ^{1}H-NMR spectrum of **17** (400 MHz, $CDCl_3$)

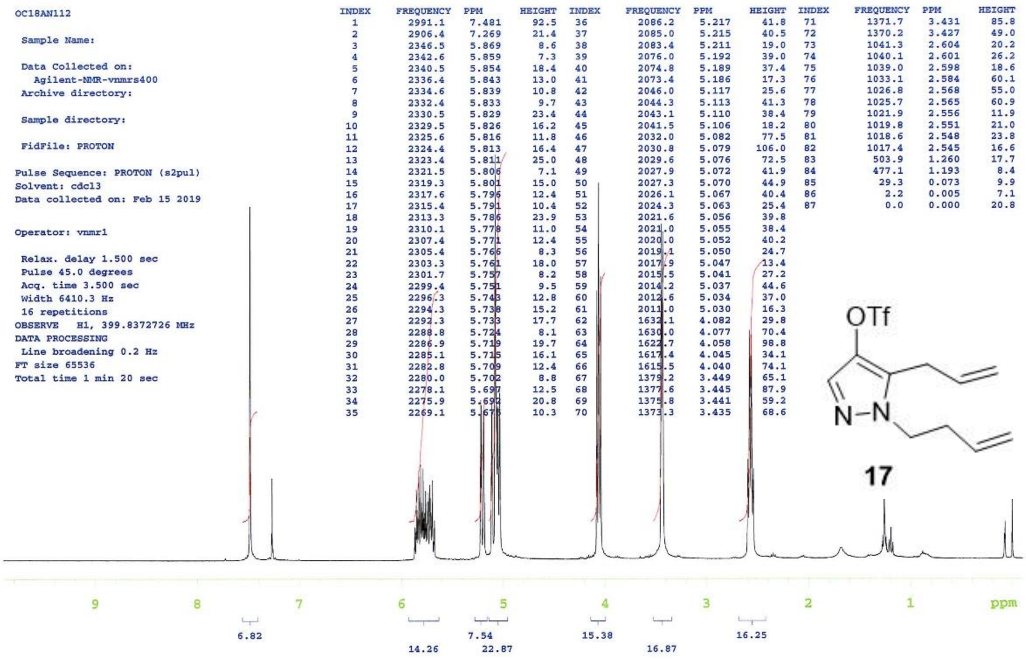

Figure S42. ^{13}C-NMR spectrum of **17** (100 MHz, $CDCl_3$)

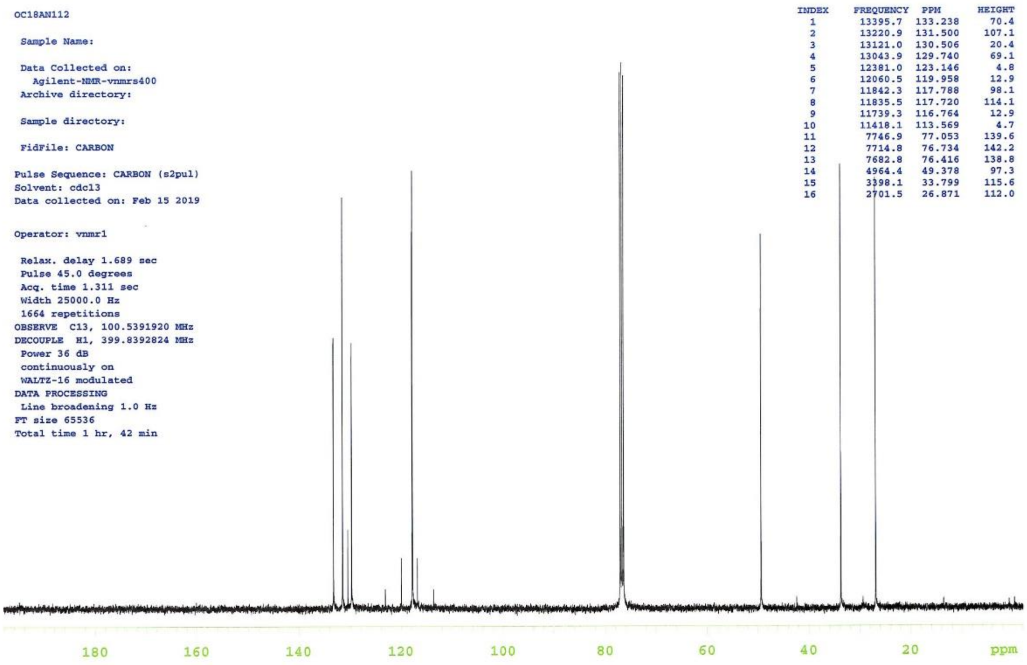

Figure S43. ^{1}H-NMR spectrum of **18** (400 MHz, $CDCl_3$)

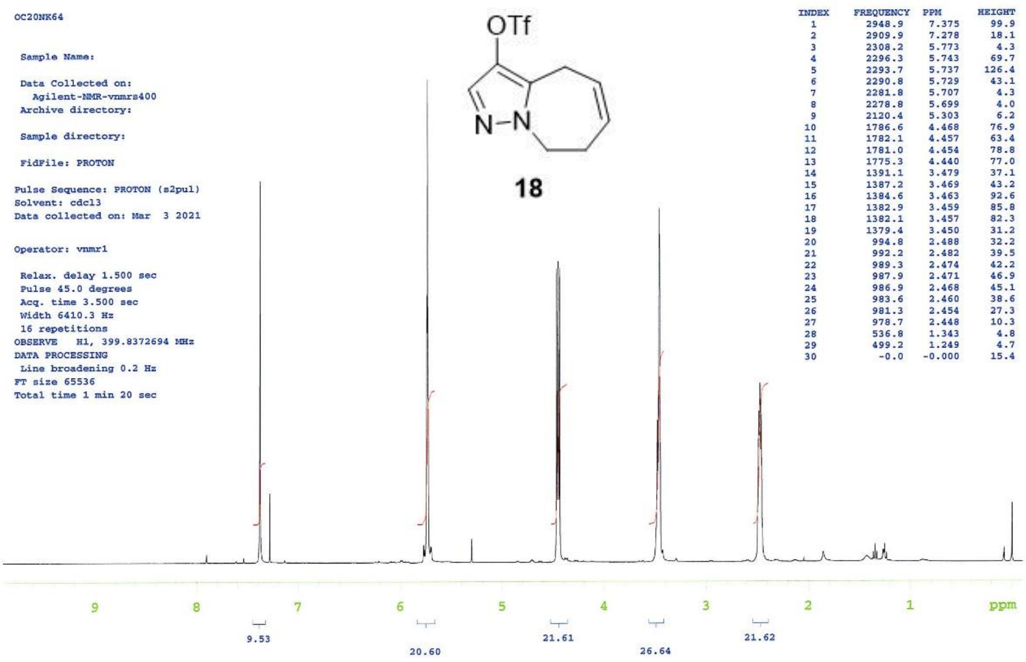

Figure S44. ^{13}C-NMR spectrum of **18** (100 MHz, $CDCl_3$)

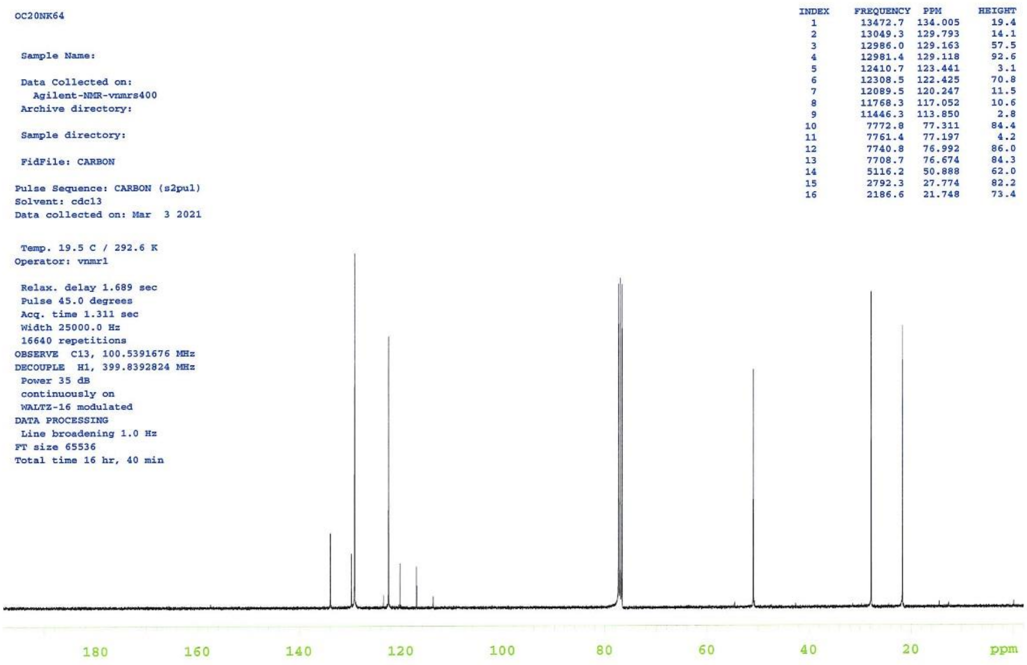

Figure S45. ^{1}H-NMR spectrum of **19** (400 MHz, $CDCl_3$)

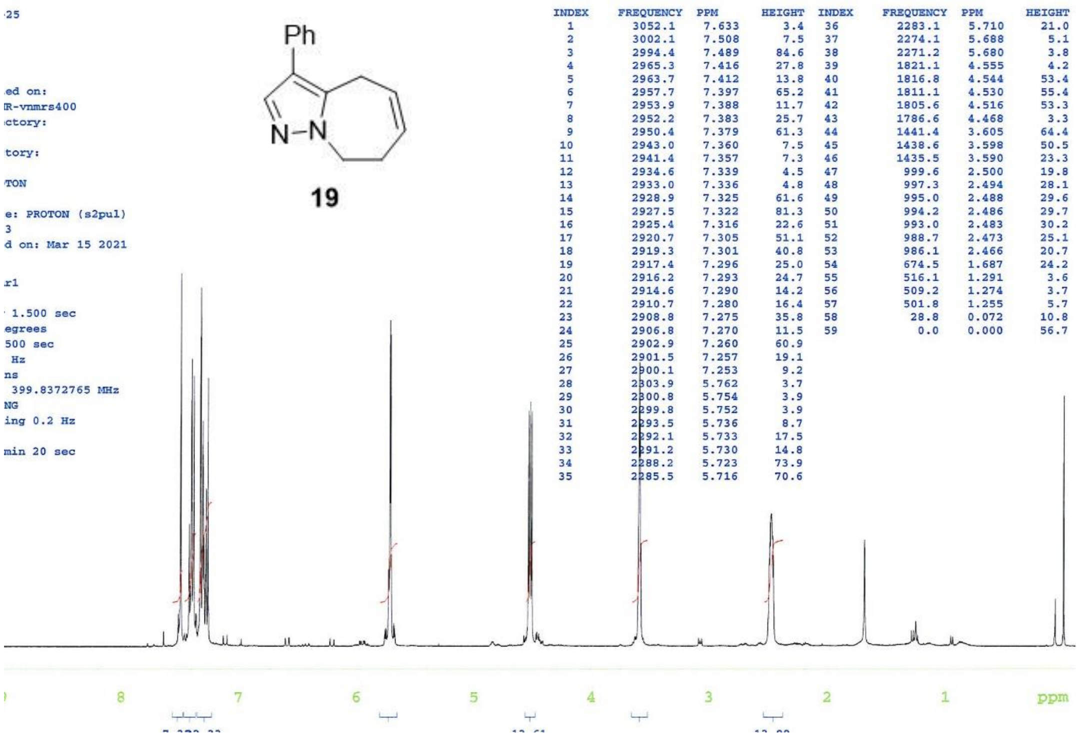

Figure S46. ^{13}C-NMR spectrum of **19** (100 MHz, $CDCl_3$)

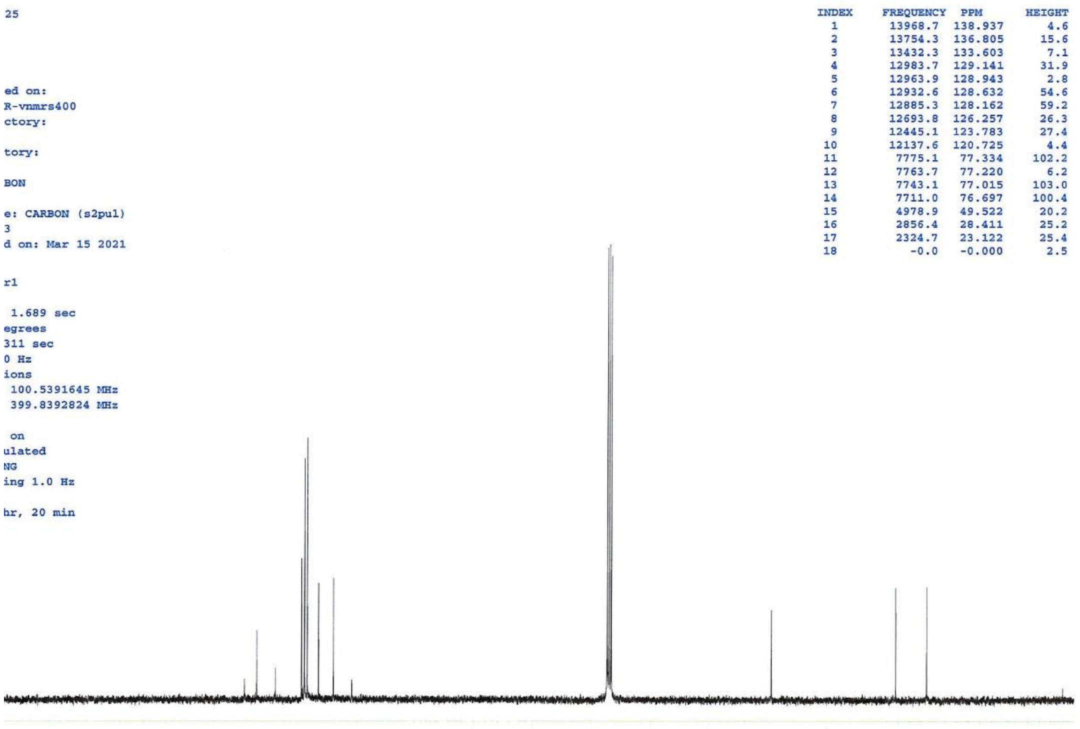

Figure S47. ^{1}H-NMR spectrum of **15** (400 MHz, $CDCl_3$)

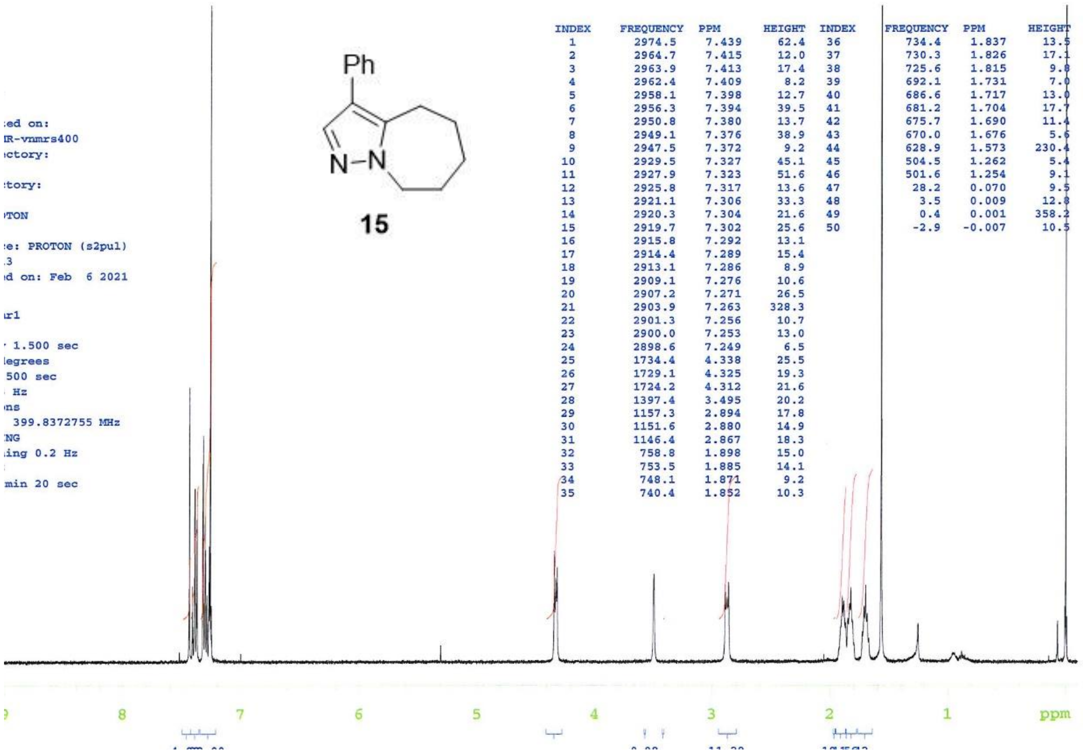

Figure S48. ^{13}C-NMR spectrum of **15** (100 MHz, $CDCl_3$)

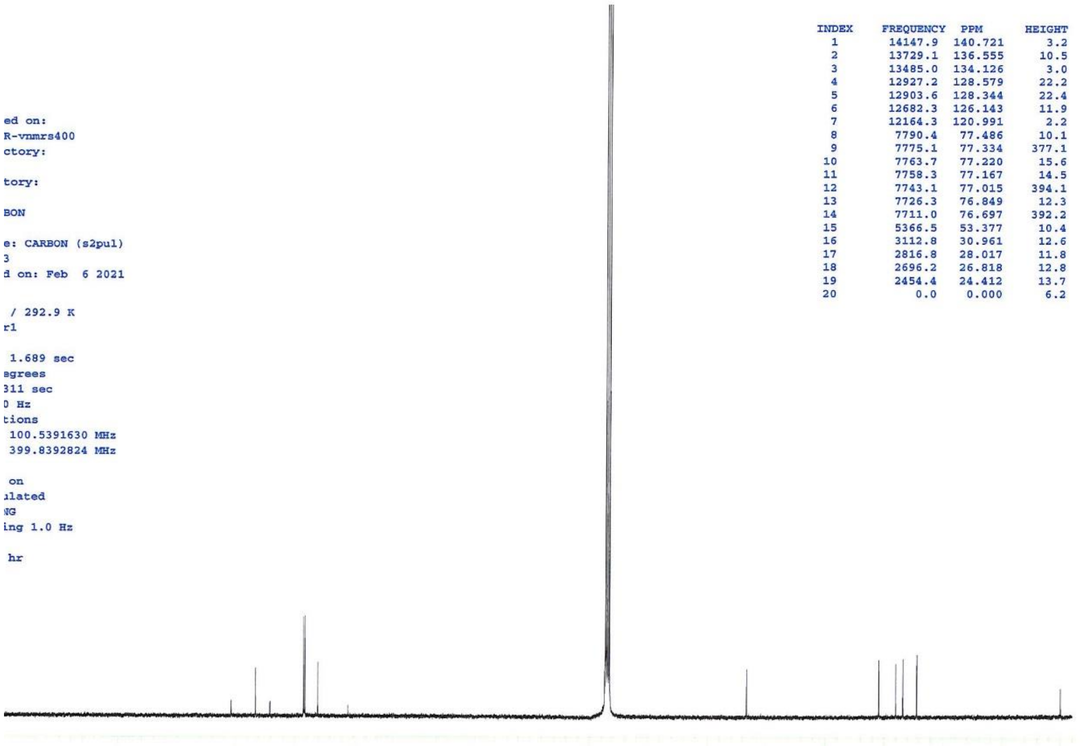

Supplement: Supplementary file 1 [file molecules-26-03370-s001.zip › molecules-1235400-supplementary.pdf]
